# Rapid ethnography and participatory techniques increase onchocerciasis mass drug administration treatment coverage in Benin: a difference-in-differences analysis

**DOI:** 10.1186/s43058-023-00423-5

**Published:** 2023-04-26

**Authors:** Arianna Rubin Means, N’koué Emmanuel Sambiéni, Euripide Avokpaho, Abdoulaye Benon Monra, Fifamè Aubierge Eudoxie Kpatinvoh, Kevin Bardosh, Moudachirou Ibikounlé

**Affiliations:** 1 Department of Global Health, Hans Rosling Center Box 351620, 3980 15th Ave NE, Seattle, WA 98195 USA; 2 Department of Sociology and Anthropology, University of Parakou, 03BP107 Parakou, Benin; 3 Institut de Recherche Clinique du Benin, Abomey-Calavi, Benin; 4 School of Public Health, University of Washington, Seattle, WA USA; 5 Division of Infection Medicine, Edinburgh Medical School, University of Edinburgh, Edinburgh, UK; 6 Centre de Recherche pour la lutte contre les Maladies Infectieuses Tropicales (CReMIT/TIDRC), Université d’Abomey-Calavi, 01BP526 Abomey-Calavi, Benin

**Keywords:** Participatory action research, Rapid ethnography, Neglected tropical diseases, Mass drug administration, Benin

## Abstract

**Background:**

Onchocerciasis, a neglected tropical disease (NTD) that causes blindness, is controlled via mass drug administration (MDA) where entire endemic communities are targeted with preventative chemotherapeutic treatment. However, in many settings, MDA coverage remains low. The purpose of this project was to determine if engaging communities in the development of implementation strategies improves MDA coverage.

**Methods:**

This study took place in an intervention and a control commune in Benin, West Africa. We conducted rapid ethnography in each commune to learn about community member perceptions of onchocerciasis, MDA, and opportunities to increase MDA coverage. Findings were shared with key stakeholders and a structured nominal group technique was used to derive implementation strategies most likely to increase treatment coverage. The implementation strategies were delivered prior to and during onchocerciasis MDA. We conducted a coverage survey within 2 weeks of MDA to determine treatment coverage in each commune. A difference-in-differences design was used to determine if the implementation package effectively increased coverage. A dissemination meeting was held with the NTD program and partners to share findings and determine the perceived acceptability, appropriateness, and feasibility of implementing rapid ethnography as part of routine program improvement.

**Results:**

During rapid ethnography, key barriers to MDA participation included trust in community drug distributors, poor penetration of MDA programs in rural or geographically isolated areas, and low demand for MDA among specific sub-populations driven by religious or socio-cultural beliefs. Stakeholders developed a five-component implementation strategy package, including making drug distributor trainings dynamic, redesigning distributor job aids, tailoring community sensitization messages, formalizing supervision, and preparing local champions. After implementing the strategy package, MDA coverage increased by 13% (95% CI: 11.0–15.9%) in the intervention commune relative to the control commune. Ministry of Health and implementing partners found the approach to be largely acceptable and appropriate; however, there was mixed feedback regarding the feasibility of future implementation of rapid ethnography.

**Conclusions:**

Implementation research conducted in Benin, and indeed throughout sub-Saharan Africa, is often implemented in a top-down manner, with both implementation determinants and strategies derived in the global North. This project demonstrates the importance of participatory action research involving community members and implementers to optimize program delivery.

**Supplementary Information:**

The online version contains supplementary material available at 10.1186/s43058-023-00423-5.

Contributions to the literature

- Stakeholder engagement is a key element of implementation research, yet there is a dearth of examples of stakeholder engagement in the identification of determinants and conceptualization of implementation strategies, particularly in low- and middle-income countries.
- We used rapid ethnography and participatory action research in Benin to develop a five-component implementation strategy package to increase treatment coverage for onchocerciasis, a debilitating neglected disease that causes blindness. This represents a novel approach to engaging multi-level stakeholders, ranging from beneficiaries to implementers.
- These findings contribute to recognized gaps in the literature, including stakeholder engagement, strategy specification, and understanding of strategy mechanisms.

## Background

Neglected tropical diseases (NTDs) are a group of chronic, disabling infections associated with poverty and inadequate access to safe water, sanitation, and housing. Onchocerciasis, also known as river blindness, is a vector-borne NTD caused by the parasitic worm *Onchocerca volvulus* which is transmitted through bites from blackflies. Symptoms of infection include severe itching, disfiguring skin conditions, and visual impairment including permanent blindness. In 2017, over 220 million people, nearly all of whom live in Africa, were at risk of onchocerciasis and 13.6 million suffered from onchocerciasis-associated skin disease while 1.15 million suffered from vision loss [1]. The World Health Organization (WHO) recommends population-based preventative treatment, known as mass drug administration (MDA), with the drug ivermectin to all individuals living in onchocerciasis-endemic areas. MDA with ivermectin tablets is extremely effective at reducing the spread of infection, primarily through the treatment of infected individuals who are both pre-symptomatic and symptomatic. As a result, the WHO and other global partners have established an objective for eliminating transmission of onchocerciasis by 2030 through the delivery of MDA with high coverage (minimum of 80% of the at-risk population treated annually) [2].

In order to engage communities and achieve high MDA treatment coverage, NTD programs have often utilized strategies such as community-directed interventions (CDI) and engagement of volunteer community drug distributors (CDDs) or other lay health workers to lead drug delivery within their own communities and neighborhoods [3–5]. Yet, onchocerciasis MDA programs, and MDA programs for other NTDs broadly, often fail to achieve global treatment coverage targets. In 2020, only 47% of the over 200 million individuals in need of onchocerciasis treatment were reached by MDA globally [6]. Thus, within many communities with active MDA programs, there remain reservoirs of infection despite decades of MDA. This reduces the likelihood of achieving elimination and requires continued expenditure of limited resources on MDA campaign management and implementation.

Rapid ethnography and other approaches to participatory action research (PAR) have rarely been deployed within NTD implementation research. PAR is an umbrella term, encompassing a variety of participatory approaches to action-oriented research. PAR involves researchers working in partnership with community members from the initial stage of project design to the final stages of drawing conclusions and identifying appropriate next steps [7]. Rapid approaches, including rapid ethnography, are increasingly used in implementation research to generate in-depth actionable information about a disease or health program to improve delivery [8, 9]. Implementation studies using these techniques have an opportunity to build new capacities for Ministry of Health (MOH) staff who manage MDA campaigns while simultaneously developing innovative implementation strategies for attaining or maintaining high MDA coverage amongst hard-to-reach populations.

The purpose of this study was to use rapid ethnography and PAR principles to develop and test implementation strategies that can optimize MDA treatment coverage for onchocerciasis elimination in Benin, West Africa. This study involved multi-level stakeholder engagement, ranging from community members to implementation leaders, and provides evidence regarding the potential utility of rapid ethnography in routine health programming within NTD-endemic countries.

## Methods

In January 2020, the Participatory Action to increase Coverage of Treatment (PACT) study was launched in Benin to identify opportunities to increase treatment coverage using rapid ethnography and PAR principles. The objectives of the study were to (1) determine if PAR improves MDA treatment coverage, (2) evaluate the time and effort requirements of using rapid ethnography, in order to understand potential compatibility for long-term MOH use, and (3) determine the acceptability, appropriateness, and feasibility of integrating rapid ethnography into MOH NTD programs from the perspective of key stakeholders. The effectiveness of the implementation strategy package deployed was evaluated using a difference-in-differences (DID) evaluation design.

This study included four main stages. Stage 1 included rapid ethnographic data collection and analysis. Stage 2 included implementation strategy package development and implementation. Stage 3 included MDA implementation and evaluation activities. And Stage 4 included dissemination activities and MOH feedback. This study adheres to Standards for Reporting Implementation Studies (StaRI) reporting specifications [10].

### Study setting

Benin is a country in West Africa with a population of 9.95 million individuals, including nearly 6 million people in need of treatment for onchocerciasis [6]. The PACT study took place in two communes: Bembèrèkè (intervention commune) and Kandi (control commune). Bembèrèkè is located in Borgou Department in northern Benin, with a total population of 100,139 individuals [6]. Kandi is located in Alibori Department, also in the north of the country, with a total population of 136,830 individuals [6]. The two communes are approximately 110 kilometers (68 miles) from one another. These communes were selected in partnership with the MOH because of their similar urban/semi-urban contexts, population sizes, and history of similar treatment coverage. Both communes participated in 18 prior rounds of annual MDA for onchocerciasis prior to study launch, and in 2019 Bembèrèkè recorded 86% MDA coverage while Kandi recorded 87% coverage [11].

### Stage 1: Rapid ethnography and PAR activities

This study conducted rapid ethnography, which is an intensive, team-based qualitative inquiry process that uses triangulation across data sources, iterative analysis, and additional data collection to quickly develop an understanding of a situation from an insider’s perspective [12, 13]. A team of rapid ethnographers was established, led by a local social scientist and two supervisors. The rapid ethnographer team was selected based on criteria of speaking the local languages in Kandi and Bembèrèkè and having prior training in social science at an undergraduate level. All team members were from the study areas or adjacent areas. The team included three men and three women from three local ethnic groups, namely the Batombou, the Dendi, and the Fulani groups. Prior to study implementation the team participated in a 2-week training activity, including mock data collection and analysis.

**Fig. 1:**
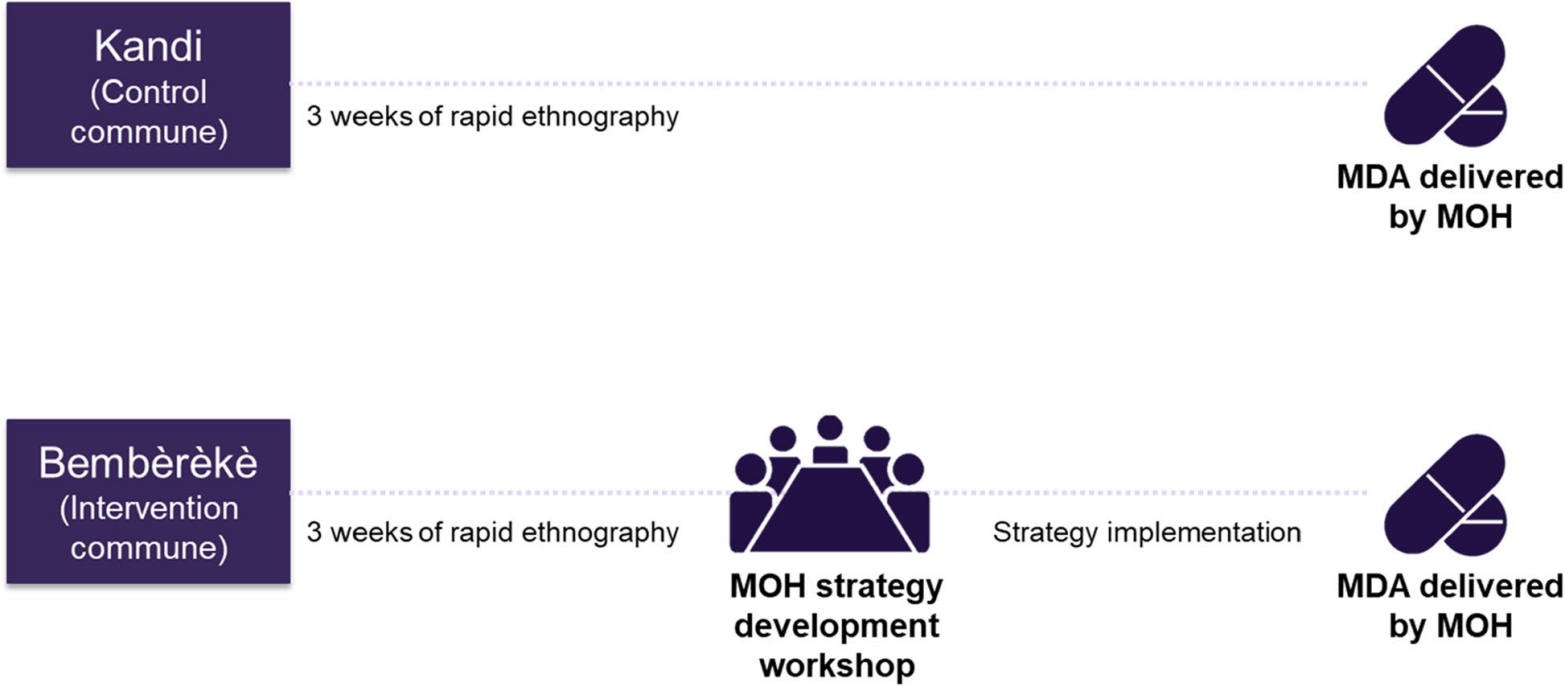
Overview of study design

A 3-week period of rapid ethnography was conducted in the intervention commune and separately in the control commune (6 weeks of rapid ethnography total) (Fig. 1). Three teams of two ethnographers engaged in approximately 14 fieldwork days in each commune, with 4 days of group analysis interspersed. The ethnographers conducted a number of data collection activities including (1) social mapping, a participatory activity used to draw maps of communities from the perspective of inhabitants; (2) daily transect walks and observations, using the social maps as guides; (3) key informant interviews with local leaders or health personnel; (4) case interviews, including individuals who did and did not participate in prior rounds of MDA for onchocerciasis; (5) informal focus group discussions with community members; (6) randomly sampled short surveys to understand prior MDA participation trends in the area; and (7) mini interviews among a subset of short survey respondents, to gain additional explanation of responses provided in the short surveys (Table 1). Study rapid ethnography tools can be found in Additional File 1.

**Table 1:** Overview of PACT study rapid ethnography tools

**Tool**	**Purpose**	**Description**	**Sample population and target sample size**
Social mapping	To identify main points of population density, MDA service delivery, and social congregation.	Upon the first 2 days of entering a commune, the research team gathered groups of local leaders together to engage in map drawing. Maps included key landmarks (ex. rivers), administrative boundaries, social services (ex. schools, clinics), centers of employment, and other notable geographic points. A composite map was created combining all maps for use in subsequent implementation strategy planning.	Maps were created by groups of 4–8 local leaders. Seven maps were created in each commune, including one at the commune level and six at the neighborhood level.
Transect walks and observation	To become familiar with the communes and communities who live there, including various social, economic, political and cultural attributes.	Rapid ethnographers moved through the community using social maps as a guide, engaging in participant observation. The teams stopped the transect walk to conduct interviews, informal group discussions or case interviews.	Three transect walks were conducted per day (one per team) in each commune, with 42 transect walks planned per commune.
Key informant interviews (KII)	To explain community perceptions of onchocerciasis, MDA, and implementation best practices from the perspective of people with special expertise or information on the topics.	KIIs were individual interviews with a purposive sample of key informants, often encountered during transect walks or during social mapping activities. Interviews were guided by an unstructured list of question prompts.	Key leaders, health workers, drug distributors, and formal and informal health workers (ex. traditional healers) were engaged in KIIs. Two KII were planned for each team per day (e.g., 6 per day and 84 total per commune).
Case interviews	To understand factors influencing behaviors of individuals who do and do not participate in prior rounds of MDA.	Case interviews were individual interviews with purposively sampled individuals who did and, separately, did not participate in the prior round of MDA for onchocerciasis. The interviews were guided by a semi-structured question guide to understand factors influencing prior behaviors, perceptions of prior programs, and plans to participate in future programs.	Two case interviews were planned for each team per day (e.g., 6 per day and 84 total per commune). The sampled population included adults in the community.
Informal focus group discussions (FGDs)	To understand group perceptions of onchocerciasis, MDA, and implementation best practices from the perspective of the general public.	Informal FGDs included a convenience sampling of groups of individuals, often encountered during transect walks. Discussions were guided by an unstructured list of question prompts.	One informal focus group discussion was planned for each team per day (e.g. 3 per day and 42 total per commune). The sampled population included adults in the community.
Short surveys	To generate a broad overview of the number of people who were reached with drugs during prior rounds of MDA and who agreed to take the drugs, by demographic group.	Short surveys included a convenience sample of individuals to conduct a two-question survey about prior treatment history. The short surveys not only provided an overview of community behaviors, but also provided the opportunity to sample for a follow-up mini interview.	Ten short surveys were planned for each team per day (e.g., 30 per day and 420 total per commune). The sampled population included adults in the community.
Mini interviews	To gain additional explanation of responses provided in the short surveys, including behavioral drivers of participation or non-participation in MDA.	Using the same principles as KIIs, mini-interviews were shorter and focused, used to clarify specific survey responses and interpretations of the research team.	Mini interviews were conducted amongst randomly sampled short survey respondents. Two mini interviews were planned for each team per day (e.g., 6 per day and 84 total per commune). The sampled population included adults in the community.

During the first week of rapid ethnography in each commune, the team aimed to learn about community perceptions of onchocerciasis and MDA. During the second week, they aimed to learn about factors influencing participation in MDA and experiences with effective or ineffective community engagement strategies. And during the third week, they aimed to learn about specific opportunities to improve implementation. Questions were tailored based on learning objective and to address key gaps in knowledge noted from the week prior. Individual rapid ethnographers maintained detailed reflective notes throughout data collection. All participants were briefed on the study purpose and provided verbal consent prior to participating in any data collection activities.

**Fig. 2:**
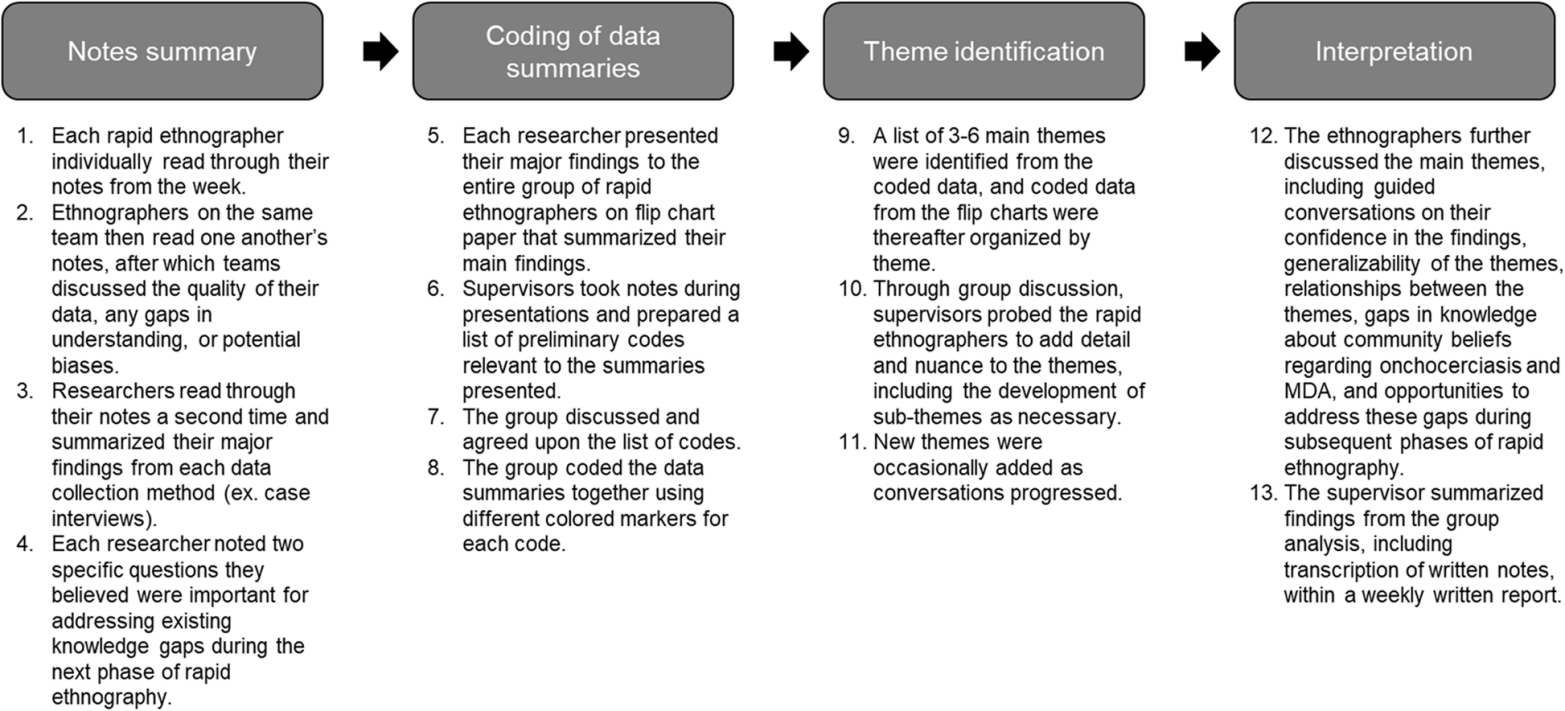
Overview of the group analysis process

Each week, a full day of group analysis took place to review data, identify main themes, and refine objectives for the following week. Each group analysis consisted of a process of individual, team, and group-based review of the data. The process involved summarizing notes, coding of data summaries, theme identification, and interpretation, as depicted in Fig. 2.

Rapid ethnographers also logged the amount of time that they spent on each data collection activity using daily paper-based time logs. A paper-based log was also developed to track the number of individuals engaged with daily by age and gender and entered into REDCap by the team supervisor. An online dashboard was created for the team to monitor and adjust their sampling strategies for the subsequent day to ensure equity in who was sampled for different data collection activities.

### Stage 2: Implementation strategy package development

A 2-day strategy development meeting was conducted in September 2020 in Bembèrèkè, Benin, to present the findings from the rapid ethnography to key stakeholders. Thirty-six individuals were invited to attend the meeting, and 34 (94%) accepted the invitation. Participants included MOH NTD personnel (*N* = 8), local leaders (*N* = 10), local health workers (*N* = 10), and community members (*N* = 6). Participants were purposively sampled in collaboration with MOH NTD leadership if they were considered critical NTD policymakers and/or implementers at national and local study levels. The purpose of the meeting was to engage stakeholders in a nominal group technique (NGT) to review findings and, as a group, derive an appropriate implementation strategy package to address observed challenges in MDA coverage, prior to the next round of onchocerciasis MDA. A NGT involves a structured approach to group brainstorming [14]. During this meeting, individuals brainstormed potential implementation strategies, took turns discussing their ideas and experiences solving similar implementation challenges, and voted on final implementation strategies to implement to address barriers and strengths observed in the rapid ethnographic data. This approach was used to ensure that the implementation strategies developed were based in the experiences of community members and were feasible for implementers.

During the meeting, stakeholders were encouraged to define implementation strategies and collaboratively describe each individual strategy’s actor, action, action targets, dose, and temporality [15]. In partnership with the PACT study team, the strategy package was thereafter implemented in the intervention commune by the MOH from September to December 2020.

### Stage 3: MDA implementation and evaluation

The MOH implemented onchocerciasis MDA from 26th December 2020 to 6th January 2021 in both the intervention and control communes. CDDs were primarily responsible for distributing treatment during door-to-door MDA campaigns, managing any adverse events, and recording treatment data using routine paper-based MOH reporting forms.

The MOH reported MDA treatment coverage data (proportion of the community treated by ivermectin), based on data collected within routine MDA paper-based treatment registers carried by CDDs. In January 2021, the PACT study team conducted an additional coverage survey in both the intervention and control communes, within 2 weeks of the final day of MDA implementation. Coverage surveys are often conducted to confirm MDA coverage and address known data quality issues, with some studies estimating that over 60% of register data are inaccurate [16, 17]. Village chiefs were informed about the coverage survey 72 hours in advance of survey launch. Each coverage survey took place over five days. The survey consisted of three sampling levels. In the first level, villages (smallest administrative unit for which a population count is available) were selected with probability proportional to size (*n* = 30). In the second stage, segments (clusters) within villages were drawn with approximately 50 households per segment, and 10 segments were selected at random. And in the third stage, 38–55 households within the segments were selected to participate based upon a pre-established sampling interval. The target sample size in each commune was 1157 households, calculated per protocol from the WHO Coverage Evaluation field guide [16, 18]. Data collectors used mobile phones and the SurveyCTO app (Dobility, Inc., Cambridge, MA, USA) to record data in the field. They also used a zippered bag containing ivermectin tablets as a prompt when asking participants if they were offered and swallowed drugs in 2019 and during the most recent round of MDA. All coverage survey participants provided verbal informed consent prior to participation. The coverage survey questionnaire can be found in Additional File 2.

A DID analysis was conducted using data from the PACT coverage survey and was used to determine if the implementation strategy package improved treatment coverage between 2019 (pre-intervention) and 2020 (post-intervention) between intervention and control communes. DID allows for the estimation of causal effects of an implementation strategy that is enacted at the group level by comparing changes over time in the intervention area with changes in the control area, assuming parallel trends (e.g., that outcomes in the intervention commune and control commune would have remained parallel over time in the absence of the PACT project) [19]. The DID analysis was performed by excluding children under five who were not eligible for treatment in either year. We conducted fixed effects linear regression using an interaction term between time and treatment group dummy variables [20]. Analyses were performed in Stata 15.1 [21].

### Stage 4: Dissemination activities

We conducted a dissemination meeting with MOH and other key partners in April 2021. The meeting consisted of a presentation of rapid ethnography findings, an overview of the strategy package deployed, and a presentation on coverage survey findings. The study team led a structured SWOT (strengths, weaknesses, opportunities, and threats) conversation with meeting participants. A survey was conducted at the end of the meeting, following the SWOT analysis, to ascertain perspectives from the attendees on whether rapid ethnography is an acceptable, appropriate, and feasible approach for increasing coverage in targeted geographic areas of Benin moving forward. Mean Likert-score survey responses are reported. The survey drew upon psychometrically validated survey items, which were adapted to the study and translated into French [22] (Additional File 3).

## Results

### Rapid ethnography findings

**Fig. 3:**
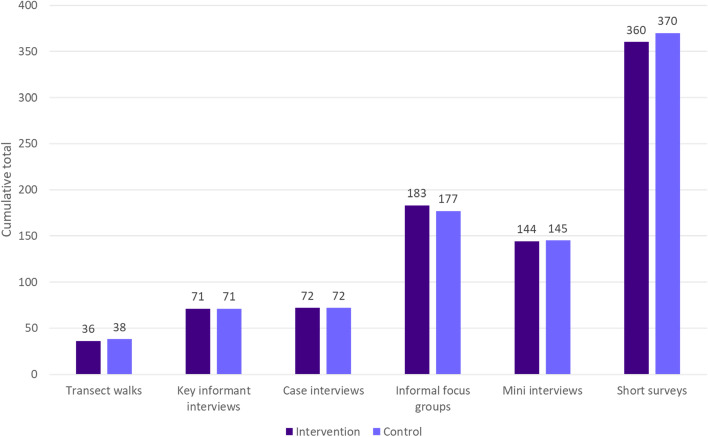
Number of data collection activities conducted, by commune

A total of 722 data collection activities took place in Bembèrèkè (intervention commune) and a total of 873 data collection activities took place in Kandi (control commune), with 1595 rapid ethnography data collection activities in total (Fig. 3). Of the 730 individuals who participated in short surveys in both intervention and control communes, only 44% and 73% reported that they were offered MDA for onchocerciasis in the past, respectively. And, among those individuals, only 34% and 30% said they chose to accept the drugs and swallow them. This demonstrated challenges related to ineffective delivery (ex. supply challenges) and low uptake (ex. demand challenges) that were further elaborated upon by other data collection activities.

Key barriers to high coverage onchocerciasis MDA identified during rapid ethnography included trust in CDDs delivering MDA (due to perceived low professionalism), poor reach of MDA programs in rural or geographically isolated areas, and low demand for MDA among specific sub-populations driven by religious or socio-cultural beliefs. These challenges are summarized within nine main themes. These themes were common across intervention and control communes.

1. Onchocerciasis awareness is not a barrier to participation

Participants in both communes were familiar with onchocerciasis and associated treatment protocols; however, there was often misinformation about the disease. Some people believed onchocerciasis is a non-contagious disease caused by hunger, bad food, uncleanliness, old age, or bewitchment and magic. There were no significant differences in community knowledge between the two communes. Thus, despite some myths and misconceptions, awareness of onchocerciasis was not a major barrier to MDA coverage in either commune.

“I am willing to take the drug because I don't want to go blind. However, no distributor has ever come to my home" – Intervention, Case interview

(2) MDA programs were perceived to be effective in reducing the spread of onchocerciasis

The vast majority of respondents in both communes emphasized that taking MDA drugs was the best way to prevent onchocerciasis and blindness, regardless of age. Most people believed that MDA is the main reason why various eye conditions have significantly reduced over the years, which has created a large degree of community confidence and goodwill in the program. The two most common reasons for taking MDA drugs were that they were free and effective. MDA was also believed to have additional health benefits beyond onchocerciasis including sexual vitality, scabies control, the prevention of insect bites, and longevity. Thus, there was generally high awareness of MDA programs in both communes, with broad receptivity amongst community members.

“I used to have problems with my eyes but since I started drinking the medication, all these problems have disappeared, the medication has been very effective” – Intervention, Case interview

(3) MDA drugs were perceived to be associated with side effects, preventing some individuals from accepting treatment

Side effects were a common reason why respondents said they refused treatment in both communes. Perceived side effects included skin allergies, itching, and vomiting. Some participants believed that side effects meant that the drugs were “waking up” diseases hidden in the body and that this could have harmful consequences. Other believed that the side effects were a positive sign of the drugs working against onchocerciasis. The way in which a person and household interpreted side effects had an important influence on willingness to participate in the MDA program. CDDs were aware that they were supposed to provide additional support to individuals who have had previous side effects from the drugs, but generally felt ill equipped to do so. These findings indicated that community members required more information about adverse events, and CDDs required more support in educating communities about drug safety.

"When some take these drugs, they become weak, vomit and sometimes go to the hospital for treatment. I think this is normal because what comes out is the diseases in their bodies" - Intervention, Focus group

(4) Compromised trust in CDDs and perceived lack of professionalism is a barrier to participation

Many participants shared that they accept MDA drugs when offered. Common exceptions included when CDDs were not representative of the ethnicity of the participant’s neighborhood or did not speak their language, when there were high levels of government mistrust, lack of trust in the distributors, or inadequate communication about MDA. Some respondents noted that CDDs would smoke, talk disrespectfully, or behave inappropriately during MDA campaigns. Respondents also reported that some CDDs did not spend much time at their households, or came during work hours when individuals were away from home. Respondents also reported several instances when CDDs refused to take the MDA drugs due to fear of side effects. This contributed to a perception in many areas that CDDs lack professionalism.

"Refusal of the drugs is sometimes linked to the behavior of distributing agents who are often in a hurry and do not explain to beneficiaries why they are distributing the drugs" – Kandi, Focus group

"We encounter a few cases of refusal and this is because communication is not getting through. [CDDs] do not explain the consequences of this disease and the benefits of this drug." – Kandi, KII

(5) Socio-cultural beliefs of some community members lead to refusals to participate in MDA

Community distributors faced various socio-cultural challenges in the field. This included myths about nefarious drug origins, norms around measurement for drug dosing, and the cultural dynamics of providing drugs across age groups. For example, many people stressed that they believe that a young adult should not measure the height of an elderly person, which is necessary for ivermectin pill dosing. This was because measuring sticks are used to gauge the size of a corpse to determine the length of a funeral pit. This information provided important evidence about cultural sensitivities that could be incorporated into CDD training.

“A lady friend came to my office one day and told me that she received a product but didn’t take it because the [CDDs] didn't say why they were giving her. For them white people are looking for ways to destroy us" – Intervention, Case interview

(6) Pre-MDA communication does not reach all neighborhoods in the catchment area

In both communes, pre-MDA communication takes place via a “town crier” who alerts communities to when treatment will occur. Participants reported that messages tended to not reach remote areas of both communes, and residents felt excluded when they did not receive information about the program. Additionally, typically mobilization begins 1–2 days before distribution and many participants noted that this was not sufficient advance warning. Some participants reported that more meetings need to be held with community leaders, including ethnic and religious leaders, to publicize campaigns. This information provided important evidence about ways to improve delivery of community sensitization to ensure there was penetration into peri-urban and rural areas of the communes.

“I have never heard of the disease. My wife is always at home, has never been visited by a distributor" – Intervention, Mini interview

"Last year distributors were not able to cover all the hamlets because the town crier didn't do his job well. He passed on the information just in center. He did not to hamlets” – Intervention, Focus group

(7) Respondents reported that drugs did not reach all areas of the communes, and many individuals were never offered treatment

Participants in several neighborhoods of both communes reported not being offered treatment in the past. In the control commune, respondents reported that nomadic Hausa groups usually did not receive MDA. Likewise, individuals in peri-urban neighborhoods reported that treatment only took place sporadically. Overall, there was a perceived degree of randomness to access based on residence as well as a lack of a systematic distribution plan to ensure that all households were reached with drugs.

"I am not satisfied because distributors did not come to my house even though it was a door-to-door" – Intervention, Case interview

"Here in [neighborhood], we don't have any information on MDA. Inhabitants of the village do not benefit from MDA" – Control, mini interview

CDDs in both communes reported challenges with delivering drugs door-to-door and the likelihood of missing houses given the lack of distribution plans. In both communes, both community members and CDDs reported that MDA drugs were often out of stock. Local leaders noted that drug stock estimates were based on old demographic data, including demographic data from prior MDA treatment registers. CDDs and leaders noted that if some neighborhoods are never reached during prior campaigns, then presumably these neighborhoods would not be in the registers, and drugs would not be allocated for them.

"As a supervisor, we don't do anything to monitor distribution. If we are told that the drugs have run out of stock, we just accept" – Control, KII

(8) In some areas, MDA was viewed as lacking legitimacy

Drugs are not administered without CDD supervision; however, respondents reported that occasionally individuals are provided medication to bring to others, which presents challenges in tracking treatment coverage. Respondents also reported that, in some areas, local political delegates work with CDDs as they visit individual homes and would also go house-to-house to ask people if they had participated. However, in other areas, there was very little involvement of religious, political, and administrative officials. There was a strong sentiment that social officials should be further engaged to lend legitimacy to MDA delivery.

“Distributing agents do the distribution in one day at the most. The rest of the time they sit under sheds or somewhere in the village and when someone comes by, they [call out] and give them the tablets" – Kandi, Mini interview

(9) CDD and NTD staff burnout was high, leading to work force challenges

Some CDDs reported that they have not been compensated for their work during previous MDA campaigns, leading to demotivation and burnout. Many CDDs felt that the incentives provided were not sufficient given the time they spend on the program. Both CDDs and CDD supervisors reported that trainings were outdated and often did not provide CDDs the skills that they needed to perform their jobs. They also reported a lack of supervisors available to support CDDs during distribution. Both community members and CDDs noted that training and supervision challenges made it less likely for community members to fully trust MDA programs.

“Distributors do not go to every home…I wonder if it is because the money they are given is not enough or if it is just unwillingness to serve the village” – Control, Case interview

### Time costs of rapid ethnography

**Fig. 4:**
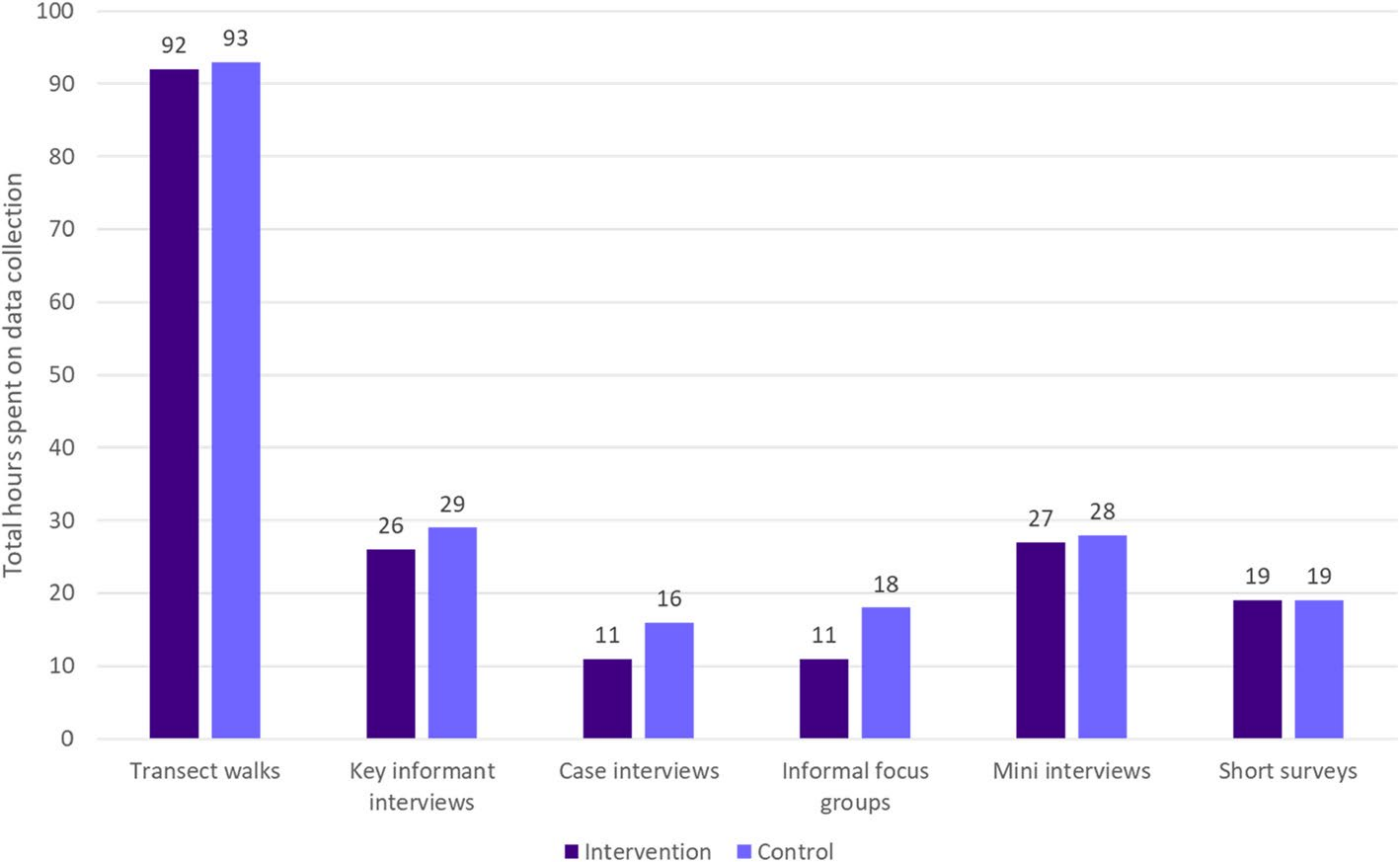
Number of hours spent on rapid ethnographic data collection, by commune

During data collection in the intervention commune, the rapid ethnography teams spent 186 hours on data collection and conducted an average of 17 data collection activities per day, per team, over a 14-day period. In the control commune, teams spent 203 hours on data collection and conducted an average of 21 data collection activities per day, per team (Fig. 4). In both the intervention and control communes, nearly half (50% and 45% respectively) of the rapid ethnography team time was spent on transect walks. Because data collection activities (e.g., mini interviews) often take place during transect walks, these specific activities are both time intensive but also necessary to facilitate ethnographic data collection.

### Strategy development and specification

Based on the rapid ethnography findings, five primary implementation strategies were identified and agreed upon during the NGT strategy design meeting. These strategies were implemented in the intervention commune, including (1) redesigning of CDD job aids to address themes about drug side effects and socio-cultural beliefs, (2) making CDD training more dynamic to address the theme of compromised trust in CDDs and perceived lack of professionalism, (3) improving CDD supervision during MDA to address themes of compromised trust in CDDs, perceptions that drugs do not reach all areas of the communes, and CDD and NTD staff burnout, (4) tailoring community sensitization/communication to address themes about pre-MDA communication and socio-cultural beliefs, and (5) preparing local champions by increasing engagement with local leaders to address the theme of MDA’s perceived lack of legitimacy in some areas. The strategies, including strategy specification details such as targeted barrier, actor, action, action target, dose, and temporality, are described in Table 2.

**Table 2:** Overview of implementation strategies deployed in the intervention commune

**Implementation strategy**	**Targeted barrier**	**Actor**	**Action**	**Action target**	**Dose and temporality**
Redesign of community drug distributor (CDD) job aids	MDA drugs were perceived to be associated with side effects Socio-cultural beliefs of some community members led to refusals to participate	PACT study team in collaboration with the MOH	The PACT study team accessed existing training manuals and worked with the MOH to redesign job aids with supportive messages that directly address community member concerns and knowledge gaps identified in the rapid ethnography. The job aids were shared with the MOH for approval and iteratively revised based upon feedback	CDDs: The strategy aimed to increase CDD knowledge and skills necessary to respond to specific points of community resistance to treatment	One redesign period took place, prior to CDD training (planning stage)
Make CDD training more dynamic	Compromised trust in CDDs and perceived lack of professionalism is a barrier to community member participation	PACT study team in collaboration with the MOH	Nearly 150 CDDs were trained in December 2020. The revised training included hands-on practice negotiating with community members about key issues that arose in the rapid ethnography. The training also highlighted topics of professionalism, including role play of ideal conduct when visiting households. We conducted pre-post tests to evaluate the improved training format, and the effects on CDD knowledge	CDDs: The strategy aimed to provide CDDs opportunity to increase knowledge and practice managing real life scenarios of treatment and community resistance to treatment in a safe training-based setting, and with peer feedback	One dynamic two-day training took place December 2020, prior to implementation (engaging stage)
Improve CDD supervision during MDA	Compromised trust in CDDs and perceived lack of professionalism Respondents reported that drugs did not reach all areas of the communes, and many individuals were never offered treatment CDD and NTD staff burnout was high, leading to work force challenges	MOH	CDDs were trained to mark the doorposts of houses with chalk during MDA, and supervisors (primarily commune-level head nurses) were trained on how to review household markings during MDA to guide feedback for CDDs. During trainings, CDD supervisors were also supported in developing new distribution maps (building on the social maps developed during rapid ethnography), to ensure entire geographic areas were not systematically excluded from MDA	CDDs: The strategy aimed to provide structured supervision necessary to ensure houses and neighborhoods were not systematically missed and build a culture of support for CDDs in their field work CDD supervisors: The strategy aimed to provide structured supervision tools with which supervisors were empowered to support and provide feedback to CDDs in specific, mutually agreed upon ways	Daily throughout 10 days of MDA implementation (executing stage)
Tailor community-level communication and sensitization	Pre-MDA communication does not reach all neighborhoods in the catchment area Socio-cultural beliefs of some community members led to refusals to participate in MDA	PACT study team in collaboration with the MOH	New sensitization messages were created for public criers and radio announcements that built upon community feedback and specific concerns noted during rapid ethnography. Public criers were trained to deliver the messages in December 2020	Community members: The strategy aimed to increase the reach of community sensitization messages and directly address community member concerns with tailored message content	In total, 61 public criers were trained on the new messages. The public criers delivered messages to the community for 5 days prior to MDA. Additionally, 30 radio spots ran daily during the two weeks prior to MDA (engaging stage)
Prepare local champions by increasing engagement with local leaders	In some areas, MDA was viewed as lacking legitimacy	PACT study team in collaboration with the MOH	Educational flyers were created for local leaders with updated guidance on how to engage community members and specific educational messages to reinforce. The flyers were shared with community leaders during a series of neighborhood-level informational meetings	Community leaders, including political, religious, and traditional leaders (ex. village chiefs): The strategy aimed to increase community leader knowledge of MDA and provide tools for leaders to support MDA implementation, increasing MDA programs legitimacy	Ten meetings with local leaders were conducted in December 2020 to distribute and discuss the educational flyers. 100 local leaders were trained within one week of the first day of MDA (engaging phase)

*CDD* community drug distributors, *MDA* mass drug administration, *MOH* Ministry of Health

The second strategy, making CDD training more dynamic, was evaluated using pre-post tests of participating CDDs. Because community members expressed concerns that CDDs were not medically qualified to administer treatment during MDA, the test focused on knowledge of onchocerciasis, ivermectin, and management of adverse events. Prior to training, 72% of the 145 CDDs who were trained did not pass the test (defined as receiving 10 out of 20 possible points, or 50% correct answers). After training, only 39% of the CDDs did not pass the test (32% reduction in failing from baseline).

### Coverage findings

**Fig. 5:**
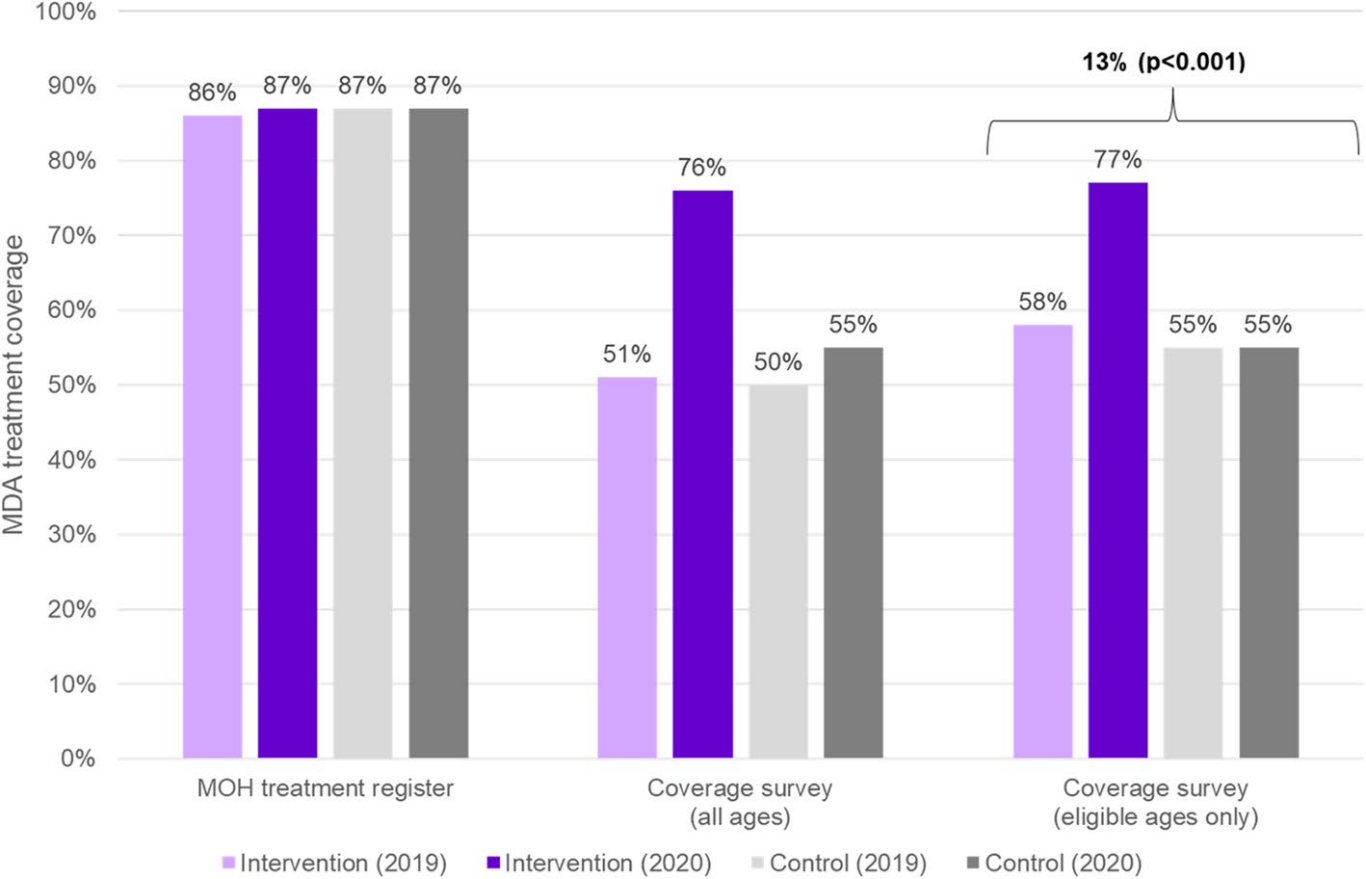
Coverage and effect of the implementation strategy package on coverage, by data source

MOH treatment registers indicated that both Bembèrèkè (intervention) and Kandi (control) achieved 87% treatment coverage during the 2020 onchocerciasis MDA. This demonstrates a 1% increase in coverage over time in the intervention commune (Fig. 5). We conducted a coverage survey to verify coverage, and to inform the primary evaluation of the intervention’s effectiveness. A total of 2,437 households participated in the coverage survey, including 14,575 individuals (8,548 in the intervention commune and 6,026 in the control commune). We found that 83% of houses were visited by CDDs in the intervention commune, while only 59% were visited in the control commune (Table 3). Additionally, 82% of individuals in the intervention commune had heard about MDA before the CDDs arrived at their home, compared to only 54% in the control commune. One of the main reasons that individuals reported that they did not receive ivermectin was that no one came to their home, and this was particularly pronounced in the control commune as compared to the intervention commune (67% versus 38%, respectively). Due to the social mobilization methods deployed, most (70%) of individuals in the intervention commune who had heard about MDA before it took place heard via the radio. The most common form of awareness in the control commune was word of mouth from family members, friends, or neighbors (46%). An equivalent proportion of individuals took ivermectin, among those who were offered treatment, in both intervention and control communes (96% in each). Amongst those who were offered ivermectin, 84% and 74% of individuals in intervention and control communes swallowed the pills directly in front of the CDD, respectively.

**Table 3:** Coverage survey results

	**Intervention *n* (%)**	**Control *n* (%)**	**Total *n* (%)**
Total households	*N = 1228*	*N = 1209*	*N = 2437*
Total individuals	*N = 8547*	*N = 6028*	*N = 14,575*
Household visited by a CDD during MDA	1020 (83.1%)	709 (58.6%)	1729 (70.9%)
Home marked by CDD^a^	910 (74.1%)	364 (30.1%)	1274 (52.3%)
Marking observed by study team	869 (70.8%)	239 (19.8%)	1108 (45.5%)
Knew about MDA in advance	1005 (81.8%)	656 (54.3%)	1661 (68.2%)
From friend/family^b^	232 (23.1%)	299 (45.6%)	531 (32.0%)
From health personnel^b^	45 (4.48%)	88 (13.4%)	133 (8.01%)
From CDD^b^	128 (12.7%)	130 (19.8%)	258 (15.5%)
From community or religious leader^b^	108 (10.7%)	97 (14.8%)	205 (12.3%)
From radio^b^	701 (69.8%)	215 (32.8%)	916 (55.1%)
From social networks^b^	11 (1.09%)	10 (1.52%)	21 (1.26%)
Other sources^b^	79 (7.86%)	35 (5.34%)	114 (6.86%)
Do not know^b^	15 (1.49%)	10 (1.52%)	25 (1.51%)
Offered ivermectin at home or in the community^c^	6118 (71.7%)	3160 (52.5%)	9278 (63.8%)
Reasons why ivermectin was not offerred^d^			
CDD did not come to home	895 (38.3%)	1843 (66.7%)	2738 (53.7%)
Individual was not eligible for treatment	860 (36.8%)	446 (16.1%)	1306 (25.6%)
Not home during MDA	466 (19.9%)	409 (14.8%)	857 (17.2%)
Had not heard about MDA in advance	108 (4.62%)	202 (7.31%)	310 (6.08%)
Drug stock was exhausted	86 (3.68%)	36 (1.30%)	122 (2.39%)
Other	24 (1.03%)	15 (0.54%)	29 (0.76%)
Consumed ivermectin^e^	5892 (96.3%)	3052 (96.6%)	8944 (96.4%)
Directly observed treatment^f^	4920 (83.5%)	2261 (74.1%)	7181 (80.3%)
Reasons why ivermectin was not consumed^g^			
Fear of side effects	24 (13.3%)	16 (25.0%)	40 (16.3%)
Bad taste	1 (0.55%)	9 (14.1%)	10 (4.08%)
Not sick, don’t need treatment	9 (4.97%)	7 (10.9%)	16 (6.53%)
Not enough information from CDDs	16 (8.84%)	3 (4.69%)	19 (7.76%)
The medicine does not work	0 (0%)	2 (3.13%)	2 (0.816%)
Not concerned by disease	66 (36.5%)	26 (40.6%)	92 (37.6%)
Other^h^	70 (38.7%)	8 (12.5%)	78 (31.8%)
Remember taking MDA in 2019^i^	3472 (50.8%)	2383 (49.9%)	5855 (50.4%)

^a^Amongst all households visited

^b^Amongst those who were aware MDA would take place before CDDs arrived

^c^Amongst all individuals surveyed

^d^Amongst individuals reporting that they were not offered ivermectin

^e^Amongst individuals offered ivermectin

^f^Amongst individuals reporting that they swallowed ivermectin

^g^Amongst individuals who refused to take the treatment when offered

^h^While responses to “other” were diverse, many respondents answering with “other” indicated that they did not take the treatment when offered because they did not believe that they were eligible and should not have been offered treatment to begin with (ex. they were sick, pregnant, etc.)

^i^Amongst individuals present to answer without proxy

In the coverage survey, an equivalent proportion of individuals of all ages (51% in the intervention commune and 50% in the control commune) reported taking ivermectin during the previous round of MDA in 2019. When limited just to individuals who were eligible (over age 5 in 2019), we observe 58% coverage (3,423 of 5,876 individuals) in the intervention commune and 55% coverage (2,291 of 4,173 individuals) in the control commune.

Coverage in 2020 is calculated as the proportion of individuals over the age of five treated, among those who were eligible. In the intervention commune, 77% of eligible individuals were treated in 2020 (5,892 of 7,687 individuals). In the control commune, 55% of eligible individuals were treated (3,052 of 5,582 of individuals). DID analyses indicated a significant increase (13.4%, 95% confidence interval:11.0–15.9%) in coverage attributable to the intervention (*p* < 0.001) (Fig. 5).

### Feasibility of implementing rapid ethnography

**Fig. 6:**
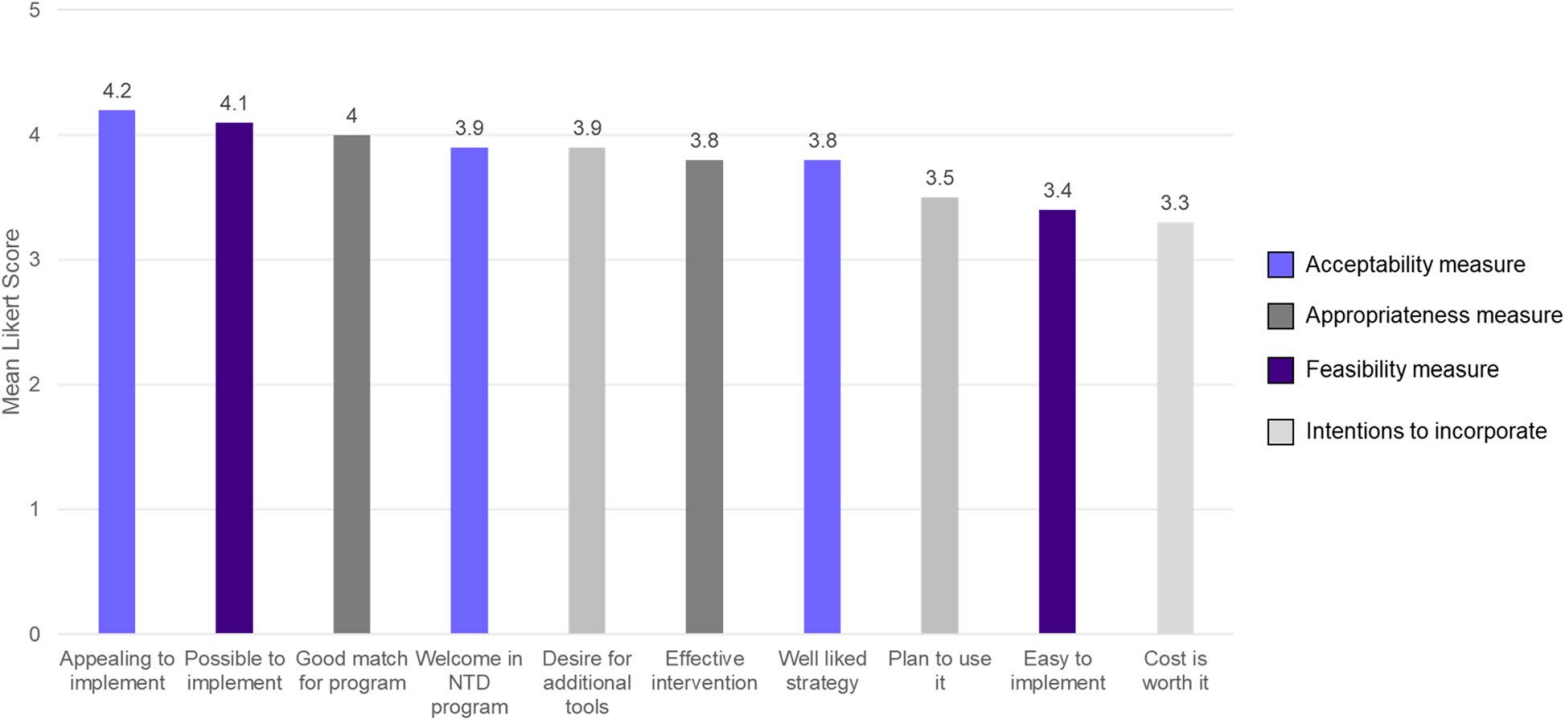
Results of acceptability, appropriateness, and feasibility assessment (*N* = 17 participants)

Seventeen individuals attended the project dissemination meeting in April 2021. After the presentation of findings, participants completed a survey in which they rated the acceptability, appropriateness, and feasibility of implementing rapid ethnography within routine MOH activities. The survey also included measures indicative of their intentions to incorporate findings into future program activities (Fig. 6). Mean response scores were highest for a measure of acceptability (4.2), indicating that rapid ethnography is an appealing intervention to implement to increase MDA coverage in Benin. However, scores were lowest (3.5 or lower) for three measures: two measures of intention to incorporate and one feasibility measure.

## Discussion

The PACT study included four main stages of implementation, starting with rapid ethnography to understand challenges related to implementation of onchocerciasis MDA from the perspective of community members, CDDs, and local leaders. During this stage we learned about key barriers to achieving MDA treatment coverage; many barriers echoed MDA delivery challenges noted in other settings, including mistrust in programs and a desire for communication to be tailored to the specific concerns of beneficiaries [23, 24]. Rapid ethnography findings also highlighted the importance of improving implementation planning and supervision to ensure all neighborhoods were reached with pre-MDA communication and treatment. Challenges with reach were highlighted by coverage survey findings, where fewer community members in the control commune heard about MDA before it took place and were offered treatment, compared to the intervention commune.

In the second phase of the study, a participatory meeting with a wide range of stakeholders resulted in the development of a five-component implementation strategy package. Because strategies are often not well matched to context [25], the purpose of this meeting was to ensure that the strategies directly addressed community members concerns noted in the rapid ethnography. Stakeholder engagement is an essential element of implementation research and is particularly important for subverting or avoiding inequities in program delivery [26]. By quantitatively tracking engagement metrics throughout rapid ethnography, we were able to self-correct when oversampling one demographic group and increase the likelihood that the rapid ethnographic findings, and the strategies they informed, were representative of the engaged communities.

In this study, we found that the strategy package resulted in more households successfully visited by CDDs and offered treatment in the intervention commune, as compared to the control commune. By specifying the implementation strategies [15], we aimed to understand not only if the package was effective but also the potential mechanisms by which the package exerted an effect. These findings suggest that the strategy package significantly increased treatment coverage in the intervention commune, primarily by increasing the reach of service delivery into historically untreated areas. This is further supported by the coverage survey observation that, once offered treatment, a similar proportion of individuals in both intervention and control communes accepted treatment. In other words, the reach of drugs improved in the intervention commune, while demand was high in both settings.

In the fourth stage of the project, MOH officials and partners attending the study dissemination meeting indicated that rapid ethnography is appealing and perhaps a good match for NTD programs. Despite the observed success of rapid ethnography and PAR for increasing MDA treatment coverage, MOH officials expressed hesitancy to adopt rapid ethnography as a strategy to increase coverage of other health campaigns. The officials were particularly unsure if the costs of the approach were “worth it”. Embedded time tracking in this study indicated that at least 389 hours (16 days) of research team time were needed to collect ethnographic data in both communes. While rapid ethnography is considered well suited for health and development sectors where resources are limited [12], the approach may need to be further simplified such that it could be operationalized during more routine campaign programming [27]. Routinizing rapid ethnography to solve coverage challenges may also require more intentional embedding of research capacity within health departments [28].

The PACT study was inherently participatory, and thus a major strength of this approach was the successful engagement of community members, the MOH, and implementing partners throughout the project. However, there were also several limitations. The most significant limitation was implementation of the project during the COVID-19 pandemic; although COVID-19 transmission did not emerge as a primary concern of community members or government officials, it is possible that the barriers and opportunities observed during the pandemic may not be representative of implementation generally. Additionally, while children under five were excluded from coverage estimates, individuals who are pregnant, breastfeeding, or sick should also not be treated with ivermectin. Because this information was not available at an individual-level pre-intervention (2019), these individuals were not excluded from the 2020 coverage calculation to avoid non-differential misclassification. Future applications of study methods would be strengthened by deploying rapid ethnography during MDA to identify opportunities to adapt and optimize implementation in real time, thereby maximizing opportunities to increase coverage.

## Conclusion

We found that using rapid ethnography and PAR to develop an implementation strategy package significantly increased onchocerciasis MDA treatment coverage. This suggests that the approach can be used to successfully identify challenges to effective MDA delivery and address those challenges using stakeholder-conceived and fit-for-purpose implementation strategies. We hypothesize that the strategy increased program reach to previously untreated areas where demand for program services was already high. Future efforts should be made to further simplify and routinize rapid ethnography and PAR approaches to increase the accessibility of these approaches and improve coverage of community-based public health campaigns.

## Supplementary Information

**Additional file 1.** Study rapid ethnography tools.

**Additional file 2.** Coverage survey

**Additional file 3.** Dissemination meeting survey

## Data Availability

The datasets generated and/or analyzed during this study are not publicly available because data analyses are ongoing on the coverage survey data for other research purposes. The coverage survey data and rapid ethnography field notes are available from the corresponding author on reasonable request.

## Abbreviations

CDI: Community directed intervention
CDD: Community drug distributor
MDA: Mass drug administration
MOH: Ministry of Health
NGT: Nominal group technique
NTD: Neglected tropical disease
PAR: Participatory action research
SWOT: Strengths, weaknesses, opportunities, threats
WHO: World Health Organization
