# Supplementary material for: Rapid ethnography and participatory techniques increase onchocerciasis mass drug administration treatment coverage in Benin: a difference-in-differences analysis
Source: Implement Sci Commun. 2023 Apr 26;4:45. doi: 10.1186/s43058-023-00423-5 (PMC10132427; doi:10.1186/s43058-023-00423-5)

TOOLKIT

for Using Community Feedback to Improve Mass Drug Administration Treatment Coverage

UNIVERSITY of WASHINGTON
COR NTD
RAP Recherche Action Participative
iRCB Institut de recherche clinique Paludisme et pathologies tropicales du Bénin
UKaid from the British people
IRD Institut de Recherche pour le Développement FRANCE

TOOLKIT
For Using Community Feedback To Improve Mass Drug Administration Treatment Coverage

ACKNOWLEDGMENTS

This toolkit was created by Drs. Euripide Avokpaho, Kevin Bardosh, Moudachirou Ibikounle, Arianna Rubin Means, and Emmanuel Sambieni. The toolkit builds upon experience and expertise in applying rapid ethnography in other countries, including India and Nepal. We would like to thank Dr. Sushil Baral, Abriti Arjyal and Obindra Bahadur Chand (HERD International, Nepal) and Dr. Shweta Prasad (Banaras Hindu University, India). In Benin, special thanks and recognition are extended to Dr Wilfrid Batcho and Dr M'PO, the NTD program Coordinator and Députy-Coordinator, respectively, Pr Achille Massougbodji, the IRCB Executive Director (Host Institution in Benin site), Department of Borgou and Alibori Health Leads, Communes of Bembèrèkè and Kandi Health responsibles, local authorities and CDD members, and NTD financial and technical partners (FHI360, Sightsavers, WHO) for their support and collaborations.

This work received financial support from the Coalition for Operational Research on Neglected Tropical Diseases (COR-NTD), which is funded at The Task Force for Global Health primarily by the Bill & Melinda Gates Foundation, by the UK aid from the British government, and by the United States Agency for International Development through its Neglected Tropical Diseases Program.

COR NTD

UKaid from the British people

EXECUTIVE SUMMARY

Delivering mass drug administration (MDA) campaigns with high treatment coverage is fundamental for achieving control and elimination of neglected tropical diseases (NTDs). Challenges to delivering MDA with high coverage include supply challenges, such as challenges in reaching all eligible individuals with sensitization, mobilization, and treatment. They also include demand challenges, often driven by community beliefs or population movement.

In areas that have experienced multiple rounds of MDA with low treatment coverage, it is important to identify the unique supply or demand challenges affecting delivery in that area. Rapid ethnography is one approach that can be used to identify implementation challenges from the perspective of local community members and health workers. Rapid ethnography provides a systematic approach for learning about these challenges and identifying opportunities to overcome them within a specific implementation unit. The purpose of this toolkit is to provide guidance to NTD programs on the use of rapid ethnography to address low MDA treatment coverage in one or more implementation units. This toolkit was created specifically for use by the Ministry of Health in Benin, but can be utilized by other NTD programs to address areas with low treatment coverage.

Rapid ethnography includes several different types of data collection activities, including: social mapping, transect walks, key informant interviews, short surveys, mini interviews, case interviews, informal focus group discussions, and participant observation. This toolkit provides guidance on how to operationalize these activities and includes several sample interview guides and other tools to support their use. When NTD programs utilize rapid ethnography, they may choose to utilize all of these data collection activities, or just a subset, depending upon the scope of the problem and the time allocated for data collection.

The toolkit provides guidance for ethical entry into a community, as well as for how to accurately record data collected within the community. Unlike approaches more closely aligned with traditional research, the approaches outlined in the toolkit are designed to address NTD program priorities by maximizing the balance between rapid, informal data collection and rigorous, generalizable findings.

This toolkit also provides guidance on how to analyze data collected during rapid ethnography. A strength of this approach is that it engages local teams to collect and interpret these data as a group. This increases the likelihood that solutions for overcoming observed challenges are feasible and acceptable, grounded in community and local health worker feedback. After rapid ethnography is completed, and challenges and potential solutions are identified, results should be provided to the national NTD program. The proposed interventions that are thereafter selected to increase treatment coverage will depend upon available program budget, timing, and replicability, amongst other considerations.

TABLE OF CONTENTS

**Acknowledgments**		A
**Executive Summary**		B
**Section 1:**	**Overview**	**7**
1A.	Where Should This Toolkit Be Used?	7
1B.	Introduction to Rapid Group Ethnography	8
1C.	Building a Team for Rapid Group Ethnography	9
1D.	Overview of Field Activity Schedule	9
**Section 2:**	**Data Collection Methods**	**11**
2A.	Social Mapping	12
2B.	Transect Walks	13
2C.	Key Informant Interviews	13
2D.	Short Surveys	14
2E.	Mini Interviews	15
2F.	Case Interviews	15
2G.	Informal Focus Group Discussions	16
2H.	Other Data Collection Methods	16
2I.	Summary of Sampling Approach for Each Method	17
2J.	Sampling Approaches to Select Participants	17
**Section 3:**	**Weekly Key Topics of Inquiry**	**19**
3A.	Weekly Key topics of Inquiry	19
3B.	Additional Topics to Cover by the Supervisor	19
3C.	Moving From Key Topics to Interview Questions	20
**Section 4:**	**Ethics and Community Entry**	**21**
4A.	Entering the Community	21
4B.	Start with Social Mapping	21
4C.	Procedures for Securing Verbal Informed Consent	21
4D.	Procedures for Securing Written Informed Consent	22
**Section 5:**	**Best Practices for Data Collection**	**23**
5A.	Basics for Data Collection in the Field	23
5B.	More "Advanced Basics"	23
**Section 6:**	**Recording Data in the Field**	**25**
6A.	Field Notes	25
6B.	Finalized Notes	25
6C.	Audio Recording	26
6D.	Informed Consent Forms	26
**Section 7:**	**Conducting Group Data Analysis**	**27**
7A.	Overview of the Analysis Approach	27
7B.	Start to the Group Analysis Sessions	27
7C.	Individual Data Processing	28
7D.	Group Analysis	28
7E.	Formal Analysis and Report Writing	31
**Appendix 1:**	**Roles and Responsibilities of Team Members**	**32**
**Appendix 2:**	**Social Mapping Instructions**	**34**
**Appendix 3:**	**Case Interview Instructions and Reporting Sheet**	**36**
**Appendix 4:**	**Mini Interview and Short Survey Instructions**	**39**
**Appendix 5:**	**Interview and Group Discussion Question Bank, by Topic Week**	**41**
**Appendix 6:**	**Introduction Strategies**	**45**
**Appendix 7:**	**Sample Written and Verbal Informed Consent for Key Informant Interviews and Case Interviews**	**46**

SECTION 1: OVERVIEW

Mass drug administration (MDA) campaigns involve treating all members of a target population with safe and effective drugs. For example, school-based programs target pre-school and school-age children, while community-based campaigns may target eligible individuals of all ages in a specific catchment area. MDA is a primary strategy used in the control or elimination of neglected tropical diseases (NTDs), including lymphatic filariasis, onchocerciasis, schistosomiasis, soil-transmitted helminthiasis, and trachoma. In order to effectively control or interrupt transmission of NTDs, MDA needs to be delivered with high treatment coverage over several campaign rounds. However, there are a number of challenges that can compromise MDA coverage. These challenges can be related to community demand for treatment, such as widespread mistrust of drugs or rumors that reduce community confidence in campaigns. These challenges can also be related to delivery of treatment, such as planning or training challenges. This manual includes guidance to support local governments and other implementing stakeholders to identify these delivery and/or demand challenges, and design effective strategies for increasing MDA coverage.

1A. WHERE SHOULD THIS TOOLKIT BE USED?

The purpose of this guide is to provide information and instructions to researchers, Program Managers, and Ministries of Health (MOH) to engage communities in activities to increase coverage of campaigns, including MDA for NTDs. The information included in this guide details a methodology to systematically solicit community feedback, so that interventions can be tailored to the specific beliefs and concerns of community members in low-coverage areas. This guide builds upon findings from the Benin Participatory Action to increase Coverage of Treatment (PACT) study in 2020.

The manual uses icons for references and to highlight important information:

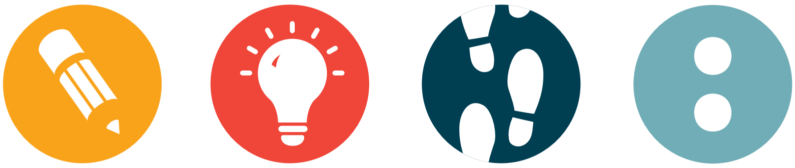

Instructions **Important** **Steps** **Definition**

1B. INTRODUCTION TO RAPID GROUP ETHNOGRAPHY

This toolkit uses a rapid group ethnography approach. "Ethnography" is a research method that aims to systematically describe social and cultural life through long-term immersion in a community. Rapid ethnography is the use of ethnography applied to a particular problem, such as low MDA coverage. Over the last few decades, rapid ethnography has been widely used in the field of education, health/nursing, marketing, and technology.

Rapid group ethnography is a team-based approach where, instead of one single researcher, a field team works together under the guidance of a senior supervisor. The approach includes:

- A team-based approach to collecting community feedback.
- A team-based approach to data analysis and interpretation.
- Fieldworkers with in-depth local knowledge of the population living in the low coverage area.

There are seven core principles of this approach that are outlined in **Box 1.**

BOX 1: 7 PRINCIPLES OF THE RAPID GROUP ETHNOGRAPHY APPROACH

Rapid group ethnography is a *team-based approach to rapid ethnographic data collection and analysis* that is led by a field team and a trained supervisor. It has seven core principles:

1. Engaging local social scientists: To address access and language barriers and build local capacity, at least half of field workers should be local to the area and have in-depth knowledge of local language, culture, and geography.
2. Flexibility and informality: The goal is to use "natural" conversations as field teams visit public areas, shops, homes, and offices to learn about MDA challenges.
3. Multiple methods: The approach triangulates (compares) a range of methods.
4. A team-based strategy: Engaging teams of field workers increases the geographical scope and social diversity of the data collection, with the goal of improving the strength and validity of the findings.
5. Group analysis: The approach uses a systematic and guided process of group analysis led by a supervisor.
6. An iterative approach: The approach moves from data collection to analysis and back to data collection to improve evidence and identify solutions for increasing coverage.
7. **Rapid results: The approach supports near real-time analysis and data sharing, with the goal of supporting rapid program planning and decision-making.**

1C. BUILDING A TEAM FOR RAPID GROUP ETHNOGRAPHY

For an administrative unit of roughly 10,000 to 20,000 people, the field team should include six field workers and one supervisor. When building a team to conduct rapid group ethnography, careful consideration must be paid to the composition of the group. The team should engage field workers with prior experience in qualitative research; a more experienced team will be able to collect higher quality data. Ideal field worker skills include a strong natural flow of conversation, strong observation skills, detailed note-taking, and analytical capability. Roles and responsibilities of each member of the team are provided in **Appendix 1.**

1D. OVERVIEW OF FIELD ACTIVITY SCHEDULE

Field workers should work in pairs. For a group of six field workers, the group should be divided into three field teams of two field workers each, and a supervisor. For a team of six field workers (3 field teams):

- Field activities should take place over a three week time period in a low-coverage area. This three-week period includes days for data collection, interspersed with days set aside for group analysis.
- Each team will explore the same research questions and use shared methods, however each team will largely collect their own data.
- Daily team meetings will be used to write notes, discuss results, and ensure data quality.
- Group analysis will occur at set intervals (e.g., biweekly) and will guide the design of each subsequent phase of fieldwork.

The approach is ambitious and requires the team to work for 5-6 days a week for three weeks in a row. It is important that the team maintain a balance between work and healthy mental and physical wellbeing. A sample field schedule can be found in **Table 1.**

TABLE 1. SAMPLE FIELD SCHEDULE FOR RAPID GROUP ETHNOGRAPHY

<table>
<tr><th></th><th>Monday</th><th>Tuesday</th><th>Wednesday</th><th>Thursday</th><th>Friday</th><th>Saturday</th><th>Sunday</th></tr>
<tr><td>Week 1</td><td colspan="2">Social Mapping</td><td colspan="3">Data Collection</td><td>Group Analysis</td><td>Day Off</td></tr>
<tr><td>Week 2</td><td colspan="4">Data Collection</td><td>Group Analysis</td><td colspan="2">Days Off</td></tr>
<tr><td>Week 3</td><td colspan="4">Data Collection</td><td colspan="2">Group Analysis</td><td>Day Off</td></tr>
</table>

BOX 2: EXAMPLE OF RAPID ETHNOGRAPHY TO INCREASE ONCHOCERCIASIS MDA COVERAGE IN BENIN

Rapid ethnography was used to increase coverage for onchocerciasis MDA in Benin. In a sample size of six neighborhoods, the team was divided into three field teams (Team 1, 2, and 3). During the three-week rapid ethnography period, there were roughly 14 fieldwork days for data collection and 4 days of analysis. The first two days were spent conducting social mapping, followed by 12 fieldwork days that combined the various methods outlined below.

SECTION 2: DATA COLLECTION METHODS

Rapid group ethnography includes periods of data collection, interspersed with data analysis **(Figure 1).** Several methods can be used to collect data for rapid group ethnography. Different methods will be used at different times in the data collection process. This section contains a brief summary of each method, and key considerations when applying the methods.

When using any method, it is important for the field teams to maintain careful notes and observations. Observations include observing community norms, interpersonal dynamics, and any differences between what people say they do or prefer and their actual behaviors.

BOX 3: METHODS THAT CAN BE USED FOR RAPID GROUP ETHNOGRAPHY

A. Social mapping
B. Transect walks
C. Key informant interviews
D. Mini interviews
E. Short surveys
F. Case interviews (with MDA participants and non-participants)
G. Informal group discussions
H. Other tools (photography, information education and communication (IEC) materials, recorders, etc.)

FIGURE 1: THE ITERATIVE DATA COLLECTION PROCESS

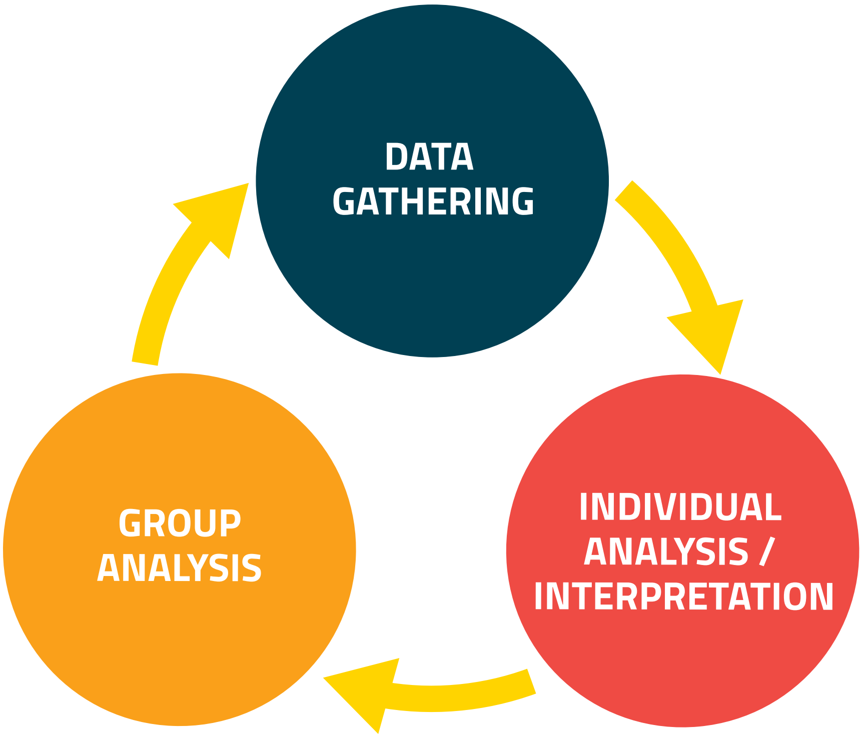

2A. SOCIAL MAPPING

Social mapping can be used early (week 1), during MDA stakeholders meeting including CDDs, health workers, and supervisors and local authorities of rapid group ethnography, to help understand a community, their experiences with MDA campaigns, and to inform subsequent sampling for other data collection activities.

During social mapping, a field team gathers groups of people (about 4-8 people) together and asks each group to draw a map of the local community. This should include key landmarks, geography, social services (e.g., schools, clinics), and occupational and socio-demographic groups.

- It is important to think carefully about who will be invited to participate in social mapping as this will determine the quality of the mapping process.
- Field team members should not participate in this mapping process; although team members should take notes about what the local mapmakers are saying during the mapping.
- After the map is done, the field team should "interview the map" and ask questions to the participants about the map.

Mapping typically takes 3-4 hours for each map. Their quality can vary. After maps have been completed, the field team and supervisor should create a composite map that combines the key aspects of multiple maps, and can aid in intervention planning. Details are provided in the social mapping instructions section **(Appendix 2).**

Sample size

Social mapping can be conducted at the district level and the neighborhood level, depending upon the low-coverage geographic area of interest. The district level social mapping should be organized and conducted by the supervisor in the first 1-2 days of field activities. Each team can then perform two social mapping exercises in different areas of the neighborhood during the first week of activities. The total number of social maps at the neighborhood level depends upon the neighborhood size.

2B. TRANSECT WALKS

Transect walks are another opportunity for learning about community members, and their experiences with MDA. They require systematically walking with local guides through the low-coverage area and observing, asking, listening, discussing, and learning from community members.

Transect walks help create an informal and casual atmosphere where the research team can ask important questions.

- Transect walks should be used to become familiar with the area and target population including various social, economic, political, and cultural characteristics of the community.
- The walks can also be used to explore the spatial dimensions of MDA delivery (asking local guides to physically point out the various MDA distribution points).
- Transect walks can also be a useful way to be introduced to community members, who may be interviewed later on.
- Transect walks can be combined with other data collection activities, such as interviews and informal focus group discussions (FGDs).

Sample size

Each field team should perform one transect walk per day (three total per day if there are three active field teams). During the walk, the team will conduct many key informant interviews, informal focus group discussions, case interviews, or other data collection activities (maintaining notes throughout).

2C. KEY INFORMANT INTERVIEWS

Key informant interviews (KIIs) are interviews with a single individual who is a key expert on the community and/or MDA campaigns in the community. These experts include *informal and formal leaders and MDA distributors (health workers, health volunteers, and teachers).*

Unstructured: Interviews that do not use a prearranged set of questions, instead, researchers ask questions on a specific topic and the interview flows like a natural conversation.

The purpose of these interviews is to understand, from an expert's perspective, community beliefs

about NTDs, MDA, and opportunities to improve MDA coverage. These interviews can also help to clarify ambiguities in data collected from community members. Each interview with a key informant should be mostly unstructured, and specific to that person, however suggested topics are included in **Appendix 5.**

Sample size

Each field team should conduct two individual interviews each day (six interviews per day if there are three active field teams).

BOX 4: WHO IS A KEY INFORMANT?

A key informant is not necessarily a formal community leader but is an expert on a specific topic. This includes people with special expertise/information about health beliefs in the community, people who can explain cultural rules and patterns, and people who have insider knowledge of how MDA is implemented in the community.

2D. SHORT SURVEYS

Short surveys can be used to collect concise feedback from a large sample size of community members. The surveys consist of very brief (2-5 questions) rapid questionnaires, often using yes/no questions. Short surveys are used prior to mini interviews (see section 2E), or other qualitative data collection techniques. For example, most short surveys ask people if they were offered treatment during prior rounds of MDA and, if so, if they took the drugs. The field team can use these responses to purposively sample a balance of individuals by age, sex, and MDA treatment history for subsequent interviews. An example short survey can be found in **Appendix 4.**

Purposively: Non-probability sampling in which field teams intentionally select individuals to participate in data collection activities.

Sample size

Each field team should conduct ten short surveys per day (30 per day if there are three active field teams).

2E. MINI INTERVIEWS

Mini interviews are conducted after a short survey, as a field team is moving through a community. The purpose of mini interviews is to answer "why" or "what next" regarding short survey responses. They are shorter and more focused than a key informant interview. Mini interviews can also be used to clarify specific questions that the field team has about their analysis findings thus far. Mini interviews are shorter and less formal than a key informant interview, and thus they can be conducted with a larger sample size of individuals. However, it may not be feasible to conduct a mini interview with all individual who participate in short surveys, and field teams should prioritize a sample that is gender balanced, includes different socio-economic groups, and engages individuals from different parts of the neighborhood (including remote areas). Further guidance is provided in short surveys/mini interview instructions **(Appendix 4).**

Sample size

Mini interviews are conducted for a subset of short survey participants. Field teams should conduct at least two mini interviews per day (six total if there are three active field teams).

2F. CASE INTERVIEWS

Case interviews are a method that can be used to generate in-depth personal information about an informant regarding their MDA-related experiences and behaviors.

Like key informant interviews, they are individual interviews. However, case interviews are focused mostly on information about the informant and their insights and perspectives on MDA. Case interviews should be semi-structured, where all individuals are asked a similar set of core questions.

Case interviews help to compare the experiences and preferences of different populations, including participants of different age groups, religions, social background, and genders. For this reason, demographic details (e.g., age, education, socio-economic status, marital status) are collected for comparison and quantitative analysis. They should always be asked at the end of the interview (not at the beginning)! An example of a semi-structured case interview guide can be found in the case interview instructions **(Appendix 3).**

Sample size

Each team should plan to conduct two case interview per day (six in total per day if there are three active field teams). Teams should try to conduct one interview with an individual who chose to participate in prior rounds of MDA and one individual who chose not to participate in prior rounds of MDA.

2G. INFORMAL FOCUS GROUP DISCUSSIONS

Informal focus group discussions (FGD) are helpful in understanding social norms around MDA participation and beliefs about NTDs. They are also helpful in understanding community ideas for improving MDA delivery. Informal focus groups are slightly different from traditional focus groups, as they do not involve pre-arranging participants in an organized fashion.

Informal FGDs are semi-structured, and field teams should be prepared with questions in advance (see **Appendix 5** for example questions).

In informal FGDs, it is okay for people to move in and out of the discussion freely. However, it is important from an ethical standpoint to inform the participants that their responses are being noted down by the field team. It is best to avoid sensitive topics in the focus groups.

Sample size

Group discussions should be conducted as a team (two field staff together). Each team should conduct one informal FGD per day.

The field team's task is to create a group of conversational partners, listening with non-judgemental interest while keeping the discussion focused and moving. Field workers should use best practices such as repeating back participant points, and asking participants if they agree with others' comments, or have anything else to add.

2H. OTHER DATA COLLECTION METHODS

Photographs: The field teams can incorporate the use of photography (via mobile phones), especially during transect walks, but also during all data collection. They should not be over-zealous, in ways that distract community members. Effort should be made to take photos to illustrate important data and findings. Always ask the community members for consent and share the photographs with them if asked.

MDA props: During interviews and group discussions, field teams can show MDA sensitization materials (e.g., flyers and posters) to community members and ask them what they think, and about their interpretation

2I. SUMMARY OF SAMPLING APPROACH FOR EACH METHOD

The sections above describe methods that can be used to collect evidence about challenges in community demand for treatment and delivery of MDA campaigns in low coverage areas. The target sample size for each method is summarized in **Table 2.** These sample sizes will vary based upon the size of the geographic area, and the number of field teams deployed as a result.

TABLE 2. SPECIFIC SAMPLING QUOTA FOR EACH TEAM

**Social mapping**	2 social maps per team per neighborhood (and 1 at the district level, conducted by supervisor).
**Transect walks**	1 per day, per field team
**Key informant interviews**	2 per day, per field team
**Informal focus group discussion**	1 per day, per field team
**Short survey**	10 per day, per field team
**Mini interview** (Extension of the short survey)	2 per day, per field team
**Case interviews**	2 per day, per field team

2J. SAMPLING APPROACHES TO SELECT PARTICIPANTS

There are a number of different sampling strategies that can be used to select participants for social mapping, key informant interviews, case interviews, informal FGDs, and short surveys/ mini interviews. Fields teams should include in their daily notes how individuals were sampled for each activity.

BOX 5: OPTIONS FOR SAMPLING PARTICIPANTS

- **Snowball sampling:** Used to locate participants by asking others to direct and introduce you.
- **Opportunistic sampling:** Taking advantage of on-the-spot opportunities.
- **Maximum variation sampling:** Attempt to speak with people who represent the full range of the sample population, to account for the full diversity of a population or group.
- **Homogenous sampling:** Selection of participants with similar characteristics.
- **Heterogeneous sampling:** Selection of participants with different characteristics.
- **Typical cases:** Selection of a typical community member.
- **Exceptions and positive deviants:** Selection of a case that is a positive deviant/exception, such as people who systematically do or do not participate in MDA.

SECTION 3: WEEKLY KEY TOPICS OF INQUIRY

3A. WEEKLY KEY TOPICS OF INQUIRY

Each week the team should have a different key topic and a set of questions to explore to understand drivers of low coverage in the geographic area. Key topics are the specific issues that need to be understood, in order to develop targeted interventions to increase coverage. Key topics to learn about each week include:

WEEK 1: COMPLIANCE AND COMMUNITY PERCEPTIONS

1. Which groups of people consistently take the MDA drugs and which groups do not?
2. What factors motivate and demotivate people from taking and swallowing the pills?
3. What positive and negative health effects do people associate with MDA?
4. How do people take the pills and what do they think about them?

WEEK 2: COVERAGE AND COMMUNITY ENGAGEMENT

1. How are different types of people involved in planning and implementing MDA?
2. What strengths and weaknesses are associated with current MDA distribution strategies?
3. What strengths and weaknesses are associated with current mobilization and education strategies?
4. What strengths and weaknesses are associated with supervision and management strategies?

WEEK 3: IMPROVING THE PROGRAM

1. How and why has the MDA program changed over time?
2. What changes in distribution strategies could improve MDA?
3. What changes in education and social mobilization could improve MDA?
4. Do people think that these changes can be made? What are the specific steps for doing so?

3B. ADDITIONAL TOPICS TO COVER BY THE SUPERVISOR

The role of the supervisor is to collect contextual data to strengthen the overall findings, as necessary. To do this, they may need to pay attention to gaps in the data. Potential questions that the supervisor may need to address during data collection include: How do people understand the biology, transmission, prevention, and control of the NTD, and what gaps in local knowledge are widespread? How is MDA influenced by power dynamics in the community (traditional power) and at the political level (local and national politicians)?

3C. MOVING FROM KEY TOPICS TO INTERVIEW QUESTIONS

As noted above, key topics of interest will evolve each week. Field teams should be prepared to adapt their data collection activities regularly as a result. Potential adaptations include:

- Preparing slightly different opening and closing statements (for different situations).
- Adapting the questions to the particular informant and situation.
- Using probing techniques to solicit more information, or to follow new ideas presented by the participant.
- Adapting interview questions as the team analyzes the data.

Probing: Questions that are designed to deepen knowledge and understanding into what someone has just told you.

A sample question bank is provided in **Appendix 5**, divided by week. This is only a sample guide; the team should adopt a more informal and conversational style in their fieldwork, and should plan to adapt these pre-determined questions based upon their experiences and findings

SECTION 4: ETHICS AND COMMUNITY ENTRY

The success of data collection activities will be dependent upon acceptability of field team activities in the community. This section provides best practices for conducting rapid ethnography in the community.

4A. ENTERING THE COMMUNITY

When first entering a community to engage in three weeks of rapid ethnography, the team supervisor should start by visiting the local authorities. During this visit, they can gather detailed information about the political, economic, and social situation in the area. It is the responsibility of the team to be professional, impartial, and politically neutral during this visit.

When entering the community and during data collection, show respect and egalitarian attitudes towards people's cultural practices, attitudes, and beliefs. Don't criticize or correct or make moral judgments.

4B. START WITH SOCIAL MAPPING

As discussed in Section 2, field activities should start with social mapping activities at both the district and local (neighborhood) levels. This will help the team build rapport with key social and political leaders. Social mapping is also a good opportunity for snowball sampling and for local leaders to make suggestions regarding who the team should speak with (e.g., MDA distributors, local officials, teachers, etc.) as key informants.

The focus of the rapid ethnography approach relies on in-depth immersion in the local socio-cultural context and participation in local activities. For this reason, it is important that field teams use various strategies to integrate themselves into the community identified via social mapping, including visits to homes, markets, or other key community gathering areas.

4C. PROCEDURES FOR SECURING VERBAL INFORMED CONSENT

Field researchers should seek verbal informed consent from all participants participating in informal FGDs or mini interviews. Start by briefly explaining in 1-2 sentences the purpose and nature of the data collection. Protect the privacy, confidentiality, and safety of the informants; do not write down their names, or disclose their names to others.

See the examples for securing verbal consent **(Appendix 7).**

4D. PROCEDURES FOR SECURING WRITTEN INFORMED CONSENT

Written informed consent may be required prior to engaging in case interviews and key informant interviews. If these activities will be audio recorded, written informed consent needs to be provided prior to turning on the audio recorder. A sample consent form can be found in **Appendix 7.**

Field workers should start by verbally explaining to the potential participant the purpose of the data collection and the risks and benefits of participation. Field workers will then read the consent form aloud to the potential participant. Then, the study staff will answer any questions that the potential participant has regarding participation. If potential participant agrees to participate, the field worker should provide them with two copies of the consent forms for him/her to sign. After the potential participant provides consent, the field worker must countersign both forms. One copy should remain with the participant and the field worker will keep the other copy. If the participant is unable to sign the consent forms because of illiteracy, they can apply a thumbprint using the inkpad. However, their name must be written on the same page by an impartial witness. The impartial witness must be an adult of the participant's choice (not the field worker) who has sat through the consenting process.

If the potential participant indicates unwillingness to participate in the interview, then the field worker should thank them, and assure them that this will not affect their access to MDA or other health services in the future.

SECTION 5: BEST PRACTICES FOR DATA COLLECTION

5A. BASICS FOR DATA COLLECTION IN THE FIELD

- Keep discussion questions simple: What? When? Who? Where? Why? How much/many?
- Avoid leading questions.
- Ask good "probes", also known as follow-up questions.
- Focus on only three or less major issues in any given interaction.
- Build "rapport" with participants, to account for social desirability bias.
- Use the language and categories of local people and avoid unnecessary biomedical terminology.

5B. MORE "ADVANCED BASICS"

Ask for consent.

- Explain the project and reaffirm the person's informed consent.

Conduct a conversation with a purpose.

- Be flexible and conversational so the participant feels comfortable speaking.
- Don't control the conversation; accept pauses.
- Be self-reflexive: pay attention to body language and what may be "hidden" or not disclosed by the participant in the interaction.

Be familiar with the questions!

- Don't look down at your notes and read questions verbatim.
- Start the conversation off with light questions, such as where are you from or how many children do you have?
- Move from general questions to more personal/individual questions once the participant is comfortable.
- Use local language and terms.
- Avoid asking too many questions; rather, use indirect methods of conversational probing.

Be prepared to probe for more information.

- Ask for clarification, examples, and details.
- Crosscheck: "I have heard..." or "I am finding that mostly men are..."
- Focus on personal stories.
- Sometimes you want to ask the same question in different ways.
- Make sure to differentiate between an individual's perceptions and practices about something and what they think others in the community think or do.
- Show "insider knowledge" to hint that you are ready for more detailed knowledge about a topic.

Take notes openly.

- Don't hide your notes. Take out your notebook when the informant says something interesting. For example, field workers can say, "What you are saying is very interesting and important. I don't want to forget parts of it. I want to get it right. Do you mind if I take some notes while you are telling me these things?"
- Use your notes to clarify and ask questions.

SECTION 6: RECORDING DATA IN THE FIELD

Capturing data is the most challenging task for the field worker. Each field workers should have two notebooks for maintaining their notes. One notebook will include rough notes from the field, while the other notebook will have finalized notes that can be used in a subsequent group analysis. *Each field worker should spend at least 1-1.5 hours each day reviewing and finalizing their notes.*

6A. FIELD NOTES

Field workers do not need to systematically audio-record data (however if the interview is particularly rich in detail or expressive, audio recordings may be taken with consent from the participant). The majority of the time, notes can be taken on small pocket-sized notebooks.

- This approach is called: "field jottings", "rough notes", or "chicken scratch".
- Retain the informants' exact words, as closely as you can. This is especially important for key phrases or creative sayings that the informant used to make an important point.
- For very important facts, you can ask: "wait a minute, this is very important for me to write down as you have just said it." Do this only for very important facts. You can read back your notes to the informant and ask them if you missed anything.
- After each discussion, or right before it ends, record (or ask) the general profile of these participant(s): gender, estimated age, social status/position.
- Notes should also include the field worker's personal thoughts about what they learned that day, as well as important questions that need to be discussed during team meetings or group analysis.

6B. FINALIZED NOTES

Field workers should spend time each day finalizing their notes in a second notebook. This moves notes from "chicken scratch" to half/full-sentence notes with descriptive details and observations. Best practices for final notes include:

- Do not only give the main highlights; describe what has been learned in detail.
- Include some verbatim quotations (in quotation marks) and local language terms.
- Don't forget the details for each interview or group discussion: Note the rough age and sex (and other relevant socio-demographic details, including social status/position) in parenthesis after a quote

While finalizing the notes, the field worker should reflect on how this new and interesting information fits into the broader group analysis and lessons learned. These ideas can also be recorded at this time.

6C. AUDIO RECORDING

Audio recordings can be collected during key informant interviews and case interviews, but only after written consent is obtained. Audio recording can be used as a form of quality control. Supervisors can do spot checks of audio recordings in order to check the accuracy and quality of field worker's finalized notes.

6D. INFORMED CONSENT FORMS

Field workers must turn in all written consent forms to the supervisor daily. These forms must be stored in a locked cabinet in a secure location. These forms are subject to auditing, and it is important that they are not lost or misplaced during field activities or transit.

SECTION 7: CONDUCTING GROUP DATA ANALYSIS

7A. OVERVIEW OF THE ANALYSIS APPROACH

Group data analysis typically involves the following steps: reading, coding data, displaying data, interpreting and refining key topics of interest for subsequent data collection **(Figure 2)**. A strength of this approach is that the analysis of data is used to inform the further collection of data.

Group analysis sessions are held biweekly, led by the supervisor. These sessions should occur after every 5 days of fieldwork. In total, 3-4 group analysis sessions should be conducted, following the schedule in **Table 1.**

FIGURE 2: STEPS OF DATA ANALYSIS

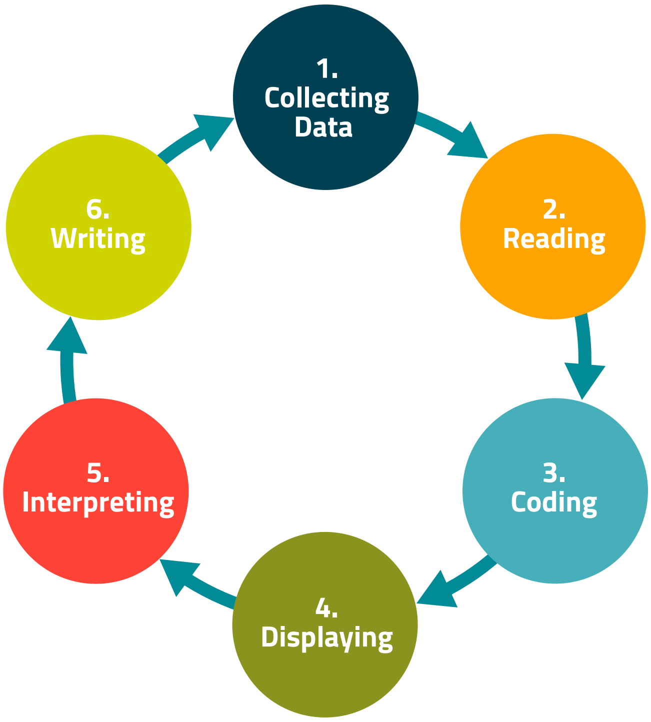

7B. START TO THE GROUP ANALYSIS SESSIONS

Each field worker should come prepared to the group analysis session with rough and final notes. At the start of the session, the supervisor will ask the group how fieldwork work proceeded this week, and the team will have a brief discussion about logistical issues and any challenges speaking with community members and rapport.

The group analysis session will then begin and can be divided into two main parts: (1) individual data processing and (2) group analysis.

7C. INDIVIDUAL DATA PROCESSING

This process should take 2-2.5 hours and will consist of Step 2 and 3 of data analysis **(Figure 2)**.

Reading (45-60 minutes)

During this step, each field worker will read through their notes to become familiar with their data in more depth. This should take 15-20 minutes.

After this, the field workers from the same field team should exchange their notes and read each other's notes. This should take about 15-20 minutes.

After they have read through each other's notes, they should have a brief conversation about the quality and depth of the data and ask: has the field worker collected meaningful and contextualized data or is the data superficial? What has been missed? They should provide constructive feedback on each other's notes and work, including any potential issues of bias. This is a chance to reflect on the quality of the data collected. The conversation should take 10-20 minutes.

Initial coding (60-80 minutes)

The field workers should then read through their own notes a second and start coding their findings. The goal is to prepare their major findings and insights for the group analysis. This will involve the following:

- Prepare a large flipchart page with the title: "major findings" and the field worker's name.
- As field workers review their notes, they should highlight with a highlighter or underline with a pen important sections of the text (e.g., important findings).
- After highlighting all important findings in the notebooks, return to the "major findings" flipchart paper. Write the number of the interview or discussion [interview 1, 2, etc.] followed by bullet points about each important finding from this interview or discussion. Do this for each data collection activity.
- Now that the field worker has a summary of major findings on one single page, think about the meaning behind them and what the data are saying about the week's key topics.
- Lastly, the field worker should note down, at the bottom of the page, two specific questions that they believe are important to learn more about in order to further understand challenges to MDA coverage, and that need to be further explored.

7D. GROUP ANALYSIS

After a break, the group analysis should take place over 4 hours and will consist of Steps 4 and 5 of data analysis **(Figure 2)**.

Displaying the data (90 minutes)

Field workers will be asked to read their flipchart page to the group. It will involve the following:

- Each field worker provides a detailed summary of their flipchart page to the group. (10 minutes max per field worker). The flipchart should be shown to the group.

- During this time, the supervisor should take notes about particular findings for the subsequent group discussion and analysis. They should also prepare a list of preliminary codes that they would like to use to code these data.
- Once all of the field workers have presented their flipchart pages, each page should be taped to the wall of the room.
- On the other side of the room, the supervisor should arrange an equal number of blank flipchart pages also taped to the wall.
- The group should then discuss the main themes that have emerged from the data so far. A consensus should be reached by all participants about the themes. There should be 3-6 main themes.
- Once this has been decided, the supervisor should write the title of each theme on each of the blank flipcharts.
- At this point, the supervisor should present to the group the preliminary codes they prepared during the presentations of the major findings. A group discussion should follow until agreement is reached on the final codes to be used to code the data. In many cases, these codes will be similar to the agreed themes.
- The group should then code all the data on the flipcharts using different colored markers **(see Box 6 below)**.
- The data should then be transferred to the blank flipcharts and organized by theme. This should occur in a way that collects similar findings together, combining them in a logical sequence. The supervisor should ask the field workers questions about the major findings to clarify and probe more deeply. Additional details should be added to the flipchart as the field workers discuss.
- If there is not perfect agreement about the relationship between a code and a theme, the supervisor should create a new flipchart page to account for the alternative coding. In this way, new themes can be added as the group discussion progresses.

BOX 6: CODING DATA

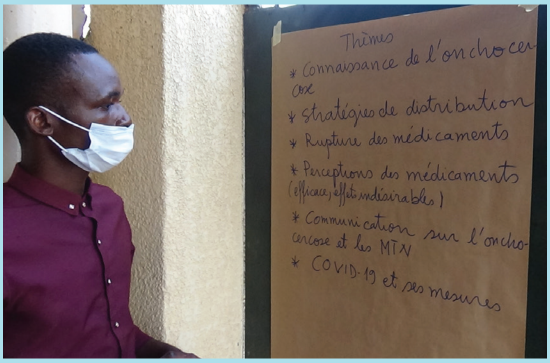

Coding is an essential part of qualitative and ethnographic research. Codes are labels of a topic or concept that is important to the study. They are words and phrases that speak for other portions of data. Think of them like street signs or different parts of a map. Codes are inserted into the margins of the text, often on the right-hand. Not every line of text is 'code worthy' and some lines may have multiple codes.

Interpreting the data (120-150 minutes)

The team should now interpret the data to identify factors influencing low MDA coverage in the geographic area, and the reasons why these factors emerge in community feedback **(see Box 7 below).** Interpreting will involve systematically moving through each flipchart page as a group following these steps:

- As themes and potentially even sub-themes are discussed, the team should ask, what can we conclude from this? Write down 1-3 major findings on a new flipchart.
- During weeks 2 and 3, the supervisor should consult findings from previous group analysis sessions to triangulate the new conclusions with findings from previous group analyses. They should read from the previous conclusions to the group, to remind them about what was discovered previously. The team should ask, how confident are we of these new findings and how they add to our previous findings? Considering these new and old findings, what information is still missing now? How should we address these gaps in our data in our field research next week?
- An important part of interpreting the data is specifying the generalizability of the conclusions: is this particular finding something that is very common, only true for a some people, or only relevant to very few people?
- Once all the flipcharts have been discussed one-by-one, the group should discuss how the major themes relate to one another. This should involve the following questions: When we think about all of the themes together, what is the data telling us about their relationships? What is missing in our understanding?
- At the end of each group analysis, the facilitator should assign specific topics for the team to focus on in the next phase of data collection. These should be decided by considering the findings and analysis in the context of the original topics of inquiry **(Appendix 5).**

BOX 7: THE *ART* OF INTERPRETATION

The 'art' of interpretation involves identifying ways that the data themes relate to one another, and what they mean in the context of understanding divers of low MDA coverage. This involves uncovering patterns and cause-and-effect relationships. Interpretation should broaden the relevance of the study to a larger population and provide answers to larger questions of social and theoretical significance.

7E. FORMAL ANALYSIS AND REPORT WRITING

It will be the responsibility of the supervisor to synthesize the data collected and analyzed over each phase of data collection into a stand-alone report. This will be done in the following steps:

1. After each group analysis session, the supervisor should collect the flipcharts and field worker notes (new notebooks may need to be distributed to field workers).
2. The next day, the supervisor should begin transcribing the data into a computer:
    - They should first transcribe all of the data from the flipcharts.
    - They should then add any notes taken during group analysis.
3. The supervisor should then summarize quantitative results (short surveys) in the report, if feasible.
4. Once this is complete, they should read through all of the field worker's notes that were prepared for the group analysis. They should add any notes of relevance to the final report. The supervisor should reach out to any individual field workers if they require clarification.
5. At the end of the document, the supervisor should note any follow-up questions and issues that were agreed upon during the last group analysis session.
6. The supervisor should have a draft of the report finished two days after each group analysis session.

The supervisor should prepare a final report that includes key findings from weekly group analyses. The final report should also include a summary of recommendations for improving coverage, which are grounded in community feedback.

BOX 8: WEEKLY REPORTS

Weekly reports are important because they represent all of the team's hard work and will be the basis of final recommendations. Producing high-quality reports will be essential. This should include sufficient use of illustrative quotes and the use of local terminology (local language terms, with appropriate translation) and it should include case studies, of particular events or people that illustrate broader findings and insights. Reports should include reflections, key findings, and next steps.

The rapid group ethnographic approach involves a team of field workers and a supervisor (ideally a senior social scientist).

Field workers should be selected to account for gender balance, prior experience with research and public health, fluency of local languages, and familiarity with local culture.

SUPERVISOR

The supervisor will take full responsibility for all scientific aspects of the fieldwork and will:

- Lead the training of the field team.
- Launch the fieldwork the week after the training.
- Create a daily calendar for the field teams and ensure that the field teams follow it.
- Collect baseline documents that can be used as conversation props, such as MDA related fliers, pamphlets, posters, radio announcements, social media, TV, and other health promotion material used by the MDA program.
- Provide supervisory support during the fieldwork period by shadowing the field teams and observing the quality of their work, especially in the first week.
- Provide feedback to the field teams daily about all methodological aspects of the fieldwork in order to improve data collection.
- Organize and lead group analysis sessions.
- Conduct social mapping at a district level at the beginning of the data collection period (week 1).
- Independently conduct targeted interviews and observations during the data collection phase to explore emerging findings and triangulate data and interpretations.
- Collect and review all coded field notes from the field workers after each debriefing session.
- Synthesize all analyses, and all primary raw data, into a full fieldwork report.

FIELD COORDINATOR (THIS ROLE IS OPTIONAL, DEPENDING UPON RESOURCE AVAILABILITY)

The field coordinator will take full responsibility for all field coordination aspects of the fieldwork and, working with the supervisor, will:

- Ensure smooth functioning of the fieldwork, including financial/administrative aspects.
- Ensure availability of transport, communication, accommodation, food, and room rental for the training and field activities.
- Coordinate with district authorities to ensure that local leaders, government officials, and senior health staff are aware and approve of the fieldwork.
- Manage all human resource issues associated with the fieldwork, including challenges with individual team members.
- Ensure the safety of the field team.
- Ensure that field teams follow the daily calendar established with the supervisor.

- Assist the supervisor with the group analysis sessions.
- Review results, as needed and requested by the supervisor, and provide input into the full fieldwork report.

FIELD WORKERS

Each field worker and field worker team will:

- Ensure full availability during the fieldwork and participate in the field training.
- Follow all details of the daily calendar established by the supervisor.
- Provide feedback during fieldwork to the supervisor daily about progress of activities.
- Ensure data collection and recording is conducted with the highest level of quality.
- Actively participate in the group analysis sessions.
- Prepare data notes prior to the group analysis sessions, as requested by the supervisor.
- Review results, as needed and requested by the supervisor.

APPENDIX 2. Social Mapping Instructions

To start, consult the key stakeholders involved in the MDA program at district and local (neighborhood) levels. Invite some social leaders, political leaders and health worker leaders that plan and implement MDA to the district-level social mapping event with sufficient advance notice.

Make sure the mapping event happens in a quiet place, and that you have a few large pieces of flip-chart paper and pens (preferably colored markers) available. Provide some refreshments.

Start by introducing the project team. Explain that this exercise is a mapping research technique, where the participants will construct a local map of the area to help reveal MDA delivery and compliance issues. Three steps should be taken for the mapping at both district and local levels.

THE STEPS FOR THE MAPPING EXERCISE INCLUDE

1) Geographical mapping

2) MDA mapping

3) Timeline

1) GEOGRAPHICAL MAPPING

This activity includes the following steps (30-45 minutes):

1. Draw and label political and administrative boundaries of the area.
2. Draw and label major roads and natural features, like rivers, farmland, forests and mountains.
3. Draw and label major urban centers (i.e., areas of high population density).
4. Draw and label any important infrastructure and industry in the area (if relevant).

2) MDA COVERAGE MAPPING.

Ask participants the following (1 hour):

5. What are the common ethnic groups here? Do they live in different parts of the area? Please mark this on the map.
6. Mark the important MDA distribution points on the map, from the most recent round of MDA for the primary NTD of interest. Both community and school MDA may need to be mapped.
7. Last year, were there areas that lacked coverage of MDA distribution points (e.g., places that lacked sufficient distribution points)? Mark these on the map.
8. Are there certain places that you think have had high MDA coverage over the prior five years? Mark these on the map.

9. Are there certain places that you think had low MDA coverage over the prior five years? Mark these on the map.
10. Ask participants if they can explain these differences (between high and low coverage), and include these in the mapping session notes.

Finally, conduct:

3) TIMELINE DEVELOPMENT

This should only be done during the district-level social mapping. Ask participants the following (1 hour):

11. Draw a timeline, from the first year of MDA for the specific NTD of interest to present day.
12. Ask what events have had an impact (positive or negative) on MDA coverage since MDA began in this area.
13. Mark these events on the timeline.

During the final group analysis session, the team should revisit the map to:

14. Update the geography, MDA coverage and timeline based upon what the team has learned over the past three weeks.
15. Highlight key points that will be important for policymakers to know, as they plan an intervention to increase MDA coverage in the area.

Case interviews are semi-structured interviews that take place with individuals who chose to accept and swallow drugs during a prior round of MDA and, separately, with individuals who did not choose to accept and/or swallow drugs during a prior round of MDA for the specific NTD of interest.

Field teams should sample individuals with different socio-economic characteristics for the case interviews, including: age, gender, ethnicity, socio-economic status, and area of residence. They should aim to balance these characteristics.

During the case interviews, the researcher should ask the following questions in a semi-structured fashion. The interview should include probes and follow-up questions. This is a guide; it should NOT be used as a questionnaire.

1. Recall the last round of MDA that you participated in, what diseases did the MDA drugs prevent or treat?
2. Did you receive MDA medications in (year) during the [disease name] MDA?
3. Did you consume MDA medications in (year) during the [disease name] MDA?
4. Why did you take/not consume the MDA medication last year?
5. Did everyone in your family consume/not consume the MDA medication last year as well?
6. Why or why not?
7. Did you know that MDA was going to happen last year before it took place?
8. About how far in advance did you hear about the MDA? How did you hear (who told you) that MDA was going to occur in (year)?
9. How did you hear (who told you) that onchocerciasis and LF MDA were going to occur in (year)?
10. Are you happy with the way the MDA drugs are distributed in this area? Why or why not (justify your response)?

At the end of the case interviews, ask the participant the following demographic details:

- Age
- Sex
- Ethnicity
- Education level
- Socio-economic status[1]
- Occupation
- Marital status
- Area of residence (rural or urban)

[1] The research team needs to standardize a locally acceptable way to classify people according to wealth category. This should not be too detailed but should be sufficient to categorize people as wealthier, middle income, poorer (according to local standards of wealth and income).

These are not the only questions you can ask the case interview participant. In fact, the interview should explore many more aspects of MDA and the motivations, perspectives, opinions and experiences of the individual. Because case interviews are semi-structured and follow a similar pattern, field workers can summarize the data collected in each case interview using the case interview data collection template below.

CASE INTERVIEW DATA SHEET

**Date:**	
**Location of interview:**	
**Name of field worker:**	
Recall the last round of MDA that you participated in, what diseases did the MDA drugs prevent or treat?	
Did you receive MDA medications in (year) during the onchocerciasis MDA? Why or why not?	
Did you consume the drugs that you received? Why or why not?	
Did everyone in your family consume/not consume the MDA medication last year as well?	
Why did your family consume/not consume the drugs for onchocerciasis during MDA last year?	
Did you know that MDA was going to happen last year before it took place? About how far in advance did you hear about the MDA?	
How did you hear (who told you) that MDA was going to occur in (year)?	

Are you happy with the way the MDA drugs are distributed in this area?	
Why or why not? (justify your response)	
Other feedback about experiences with MDA and opportunities to improve MDA coverage in this area	
Demographics	
Age	
Sex	
Ethnicity	
Educational level (last grade completed)	
Socio-economic status (low-income, middle-income, wealthy)	
Marital status	
Area of residence (rural/semi-urban/urban)	

Each field team will conduct ten short surveys per day. These short surveys provide information necessary to launch into mini interviews, for a select group of individuals. Short surveys consist of the following yes/no questions, which will be analyzed quantitatively. The questions include:

1) Last year in (year), were you offered drugs during MDA for [NTD name]?
2) Last year in (year), did you take and swallow the drugs during MDA for [NTD name] ?

Consider the short survey as a way to engage community members in a mini interview. In at least two short surveys, field teams should extend the topic of conversation. In this case, the short survey becomes a mini interview. The length of time and particular focus of mini interviews can vary widely, but should be less than 20 minutes. But it is okay for them to become much longer interviews and group discussions, if the conversation is rich. Mini interviews also provide the opportunity to learn about lingering questions and fill in gaps in information. For example, if many people have told the field team that organizing MDA in June is a bad idea because there is a local religious festival and many people are busy, or that pastoralists have migrated with all of their animals and are not available that time of year, mini interviews will allow the field team to ask many different types of people about this and quickly see how “true” this really is.

Results from the short surveys should be provided to the supervisor each day via a paper form and should be entered daily into an electronic database (e.g., Excel, ODK, etc.). An example paper form is included below.

SHORT SURVEY TRACKER (TO BE SUBMITTED DAILY)

**Date:**					
**Location of data collection:**					
**Name of field worker:**					
	**Age** *(Number)*	**Sex** *(M/F)*	**Individual was offered drugs during (year) MDA** *(Yes/No)*	**Individual swallowed the drugs received during (year) MDA** *((Yes/No/NA)*	**Individual invited for a follow-up mini interview, case interview, or focus group** *(No/mini interview/case interview /focus group)*
Individual 1					
Individual 2					
Individual 3					
Individual 4					
Individual 5					
Individual 6					
Individual 7					
Individual 8					
Individual 9					
Individual 10					

The question bank below should inform the key informant interviews and informal focus group discussions, but can also be drawn from for case interviews and mini interviews, as necessary.

WEEK 1: COMPLIANCE AND COMMUNITY PERCEPTIONS

Topics of inquiry:

1. Who participates in MDA and who does not?
2. What factors motivate and demotivate people from receiving the pills delivered during MDA?
3. What factors motivate and demotivate people from swallowing the pills delivered during MDA?
4. What positive and negative health effects do people associate with MDA?
5. How do people take the pills and what do they think about them?
6. How has COVID-19 influenced community member willingness to engage in community-wide MDA?

Potential interview questions (in no particular order):

1. Can you please describe the MDA program for [NTD name] conducted last year? What do you think about it?
2. Why do they give out this medication every year in the district? What do these medications do?
3. What is [NTD name]? How does it spread?
4. Do you know anyone with this disease or who had the disease here in this community? Do you think you are at risk of this disease? Why?
5. Did you take the pills last year? Why or why not?
6. Have you taken the pills every year? What about your family members? Your neighbors? Why or why not?
7. Do most people in this community take and swallow the pills during the MDA?
8. What types of people refuse the pills and why?
9. What do community members do with the pills if they do not swallow them?
10. What about: elderly people, children, pregnant women, people with pre-existing health conditions, certain religious groups, and migrants – do they take the pills? Why or why not?
11. What makes people more likely to take and swallow the pills? Why?
12. What makes people less likely to take and swallow the pills? Why?
13. What positive things do people say about the MDA program?
14. What negative things do people say about the MDA program?
15. Do people experience side effects from the pills? Have you heard of anyone experiencing any kind of side effects because of MDA? If yes, how do they cope with side effects? Where do they seek care?

16. Please tell me about people's current reactions to the number of pills that people are offered during MDA? Do they take them all at once?
17. Are there any cultural beliefs around the number of pills? Taste? Swallow versus chew (for albendazole)?
18. What are the health priorities and concerns of the community? How does MDA factor into these priorities?
19. Are you concerned about being infected with COVID-19 during MDA campaigns? Why or why not?
20. How has COVID-19 affected your willingness to participate in healthcare services in your community?

WEEK 2: **COVERAGE AND COMMUNITY ENGAGEMENT**

Topics of inquiry:

1. What strengths and weaknesses are associated with current MDA distribution strategies?
2. What strengths and weaknesses are associated with current MDA mobilization and education strategies?
3. What strengths and weaknesses are associated with MDA planning, supervision and management strategies?
4. How has COVID-19 influenced community member willingness to engage in community-wide MDA?

Potential interview questions (in no particular order):

1. Is MDA delivered equitably within the community? If not, who is missed and why?
2. What are particular characteristics of this neighborhood that make it particularly easy or challenging to deliver MDA?
3. How are the pills for [NTD name] distributed? Who distributes the pills? Are there any problems with how the distributions take place?
4. Are community members aware of the distribution beforehand? If yes, how many days in advance? How did you come to hear about the MDA distribution before it took place?
5. What activities have you performed for the MDA program, and for how long?
6. Are you happy with the MDA program and its implementation modality? Why or why not?
7. What training do people involved in MDA receive? Do they think it is adequate?
8. What information/counseling did the distributor provide while giving medications?
9. Do community members trust the MDA program and the people who give the pills? Why or why not?
10. Do community members take the medication in front of the person who gave the drug or not? If not, why?
11. How common is it for people to take the pills in front of the distributor? How challenging is this to carry out? What needs to be added to make this happen?

12. Generally, do you feel that the MDA program consults and engages with social leaders in the community?
13. What strengths and challenges have you seen with community engagement?
14. Do you feel that logistics and the supply of drugs and other materials have been well managed? What were not well managed and why?
15. Do the MDA distributors come from different social groups (socio-economic, age/gender, and ethnic groups)? Do they have shirts, badges, and other forms of identification? Do they have educational materials to share with people?
16. Are the MDA teams adequately staffed? Are they overburdened? How has this affected the MDA?
17. What types of data are collected during MDA? Do you face any challenges while collecting and reporting data? If yes, can you explain them with examples?
18. What types of education materials does the MDA program use? How frequently do they play radio announcements? How many people do they engage in mega-phone messaging? How many posters do they use?
19. Generally, do you feel that the MDA program does enough education and awareness activities? Are there areas of the commune that lack these activities? Why?
20. What do you think about the educational materials and messages? Are they appropriate? Do people understand them or are there misunderstandings with the language, pictures, or presentation style? Are they available in sufficient quantities and placed in enough locations?
21. Do you think the MDA program considers community perspectives and concerns in how it carries out the activities?
22. Do you think the MDA program is implemented at the best time of year, considering weather/climate and socio-cultural factors?
23. What strengths and challenges have you seen with the recruitment and management of MDA distributors?
24. Do you feel that education and social mobilization has been done adequately? If yes, how was it done? If not, why?
25. Does the MDA strengthen the health system or does it over-stretch resources and staff? How?
26. How do program staff plan the MDA? Who is primarily engaged in the planning process? How?
27. Is community feedback used to improve MDA? Is feedback from the drug distributors and community leaders used to improve MDA?
28. Should anything change about the way MDA is delivered to reduce the risk of COVID-19? How so?

WEEK 3: **IMPROVING THE PROGRAM**

Topics of inquiry:

1. How and why has the [NTD name] MDA program changed over time in the commune?
2. What changes in distribution strategies could improve MDA?
3. What changes in education and social mobilization could improve MDA?
4. Are these changes feasible for the MOH to take?
5. What measures can be taken to proactively account for COVID-19 in program design?

Potential interview questions (in no particular order):

1. How have MDA implementation strategies changed over time and why?
2. How have people's perceptions of the MDA program changed over time?
3. How might COVID-19 change people's perceptions of MDA program acceptability or safety?
4. What changes have you seen with the MDA program over the years? What is positive about these changes? What factors have played key roles in making positive changes?
5. How could community and social groups become more involved in MDA?
6. How should education material and messages be changed to improve MDA coverage in this area?
7. How should MDA delivery be changed to meet the needs of specific groups?
8. How should community mobilization be changed to improve MDA coverage in this area?
9. How can the recruitment of MDA staff be improved in order to better motivate MDA distributors?
10. What lessons could MDA programs learn from other health and social programs that operate or have operated in the district?
11. How can communities be more encouraged to take the drugs?
12. What types of pro-social behavior could be leveraged? How should this differ with different social groups?
13. Do people believe that control/elimination of [NTD name] is a feasible goal through MDA alone or are other strategies also needed?
14. How do you believe the MDA program can be improved?
15. What challenges continue to face the MDA program? Why? What are the possible reasons for persistent challenges?

"Hello. I am a field worker working with [.......] and we are asking people in the community about their thoughts and experiences regarding mass drug administration (MDA). We are asking people many different types of questions. The goal of our work is to understand how the MDA program can be improved in this community."

SAMPLE WRITTEN CONSENT

Introduction

The purpose of this consent process is to explain the purpose of this project and provide you with information to help you decide whether you would like to participate in this interview. You can ask any questions that you have about this project, including the possible benefits and risk, your rights as a participant or other clarifications.

Goal: The goal of this interview is to [insert here]

Benefits: You may find that participating in this interview may [add benefits]

Risks, stress, and discomfort:

[add any risks here]

If there are no risks, the following statement can be used:

There are no anticipated or un-expected risks or adverse events from this interview. You have the option of remaining anonymous and information will be synthesized in the analysis in a way that does not harm you or lead unwillingly to your identification. If you feel uncomfortable at any moment you can ask us to stop with the evaluation. You are also free to stop participating at any time during our visit.

Your rights and confidentiality: The information you share with us is confidential. We will not record your name, phone number, or address that could identify you. Your responses will be given a code. Your answers will be kept in a private place that only people on the project can see. We will not include your name on any reports from this evaluation.

If you agree to participate, we will ask for your signature or right thumb mark, to indicate your consent. We will give you a copy of this form to take with you.

If you ever have any questions about this project, you may contact [add contact].

Do you have questions about the project or taking part in this evaluation?

Declaration of Participant

The purpose of this interview was explained to me. I received a copy of this consent form.

I volunteer to participate in observation.

____

Participant's signature or thumb print   Date

____

Signature of witness (if needed)   Date

SAMPLE VERBAL CONSENT SCRIPT

Note: All final verbal or written consent documents should be reviewed and approved by a local ethical review committee

> **"Hello, my name is ____________ (name), and this is my colleague ____________ (name). We are working with ____________ (organization/government name) in an effort to improve delivery of mass treatment programs in this area. We would like to learn about your perspectives on the disease ____________ (NTD name) and the mass treatment programs that are used to stop the spread of ____________ (NTD name). The information you share with me will help improve these programs in the future. This ____________ (interview/discussion/survey) will take about ____________ (minutes) of your time.**
>
> **I will not record your name, to ensure that your responses are private. The anonymous responses that you share with me today will be shared with the NTD program and potentially used in a scientific report (publication) to help inform future improvements to program delivery. There is a risk that others may hear your responses while we speak and, if that is concerning to you, we would be happy to meet with you somewhere more private, such as ____________ (health facility meeting room, or other pre-arranged location). Participation in this ____________ (interview/discussion/survey) is voluntary. If you decide not to participate, there will be no penalty of any kind. You can, of course, decline to answer any of the questions we will ask, as well as stop participating at any time, without any issue.**
>
> **If you have any additional questions concerning this activity or your participation in it, please feel free to contact me or my supervisor anytime. I am giving you an informational card about this project, with my supervisor's contact details on it."**

(The data collector provides an informational card, when applicable, with the title of the project, their name, institutional affiliation, and supervisor contact information.)

If the data collector is recording the exchange they additionally add: "I would like to make a tape recording of our discussion, so that I can have an accurate record of the information that you provide to me. I will transcribe that recording by hand, and will keep the transcripts confidential and securely in my possession."

> **"Do you have any questions about this project?**
>
> **Do you agree to participate [Insert the following when applicable: 'and may I record our discussion']?**
>
> **(If yes) Thank you, let's begin..."**

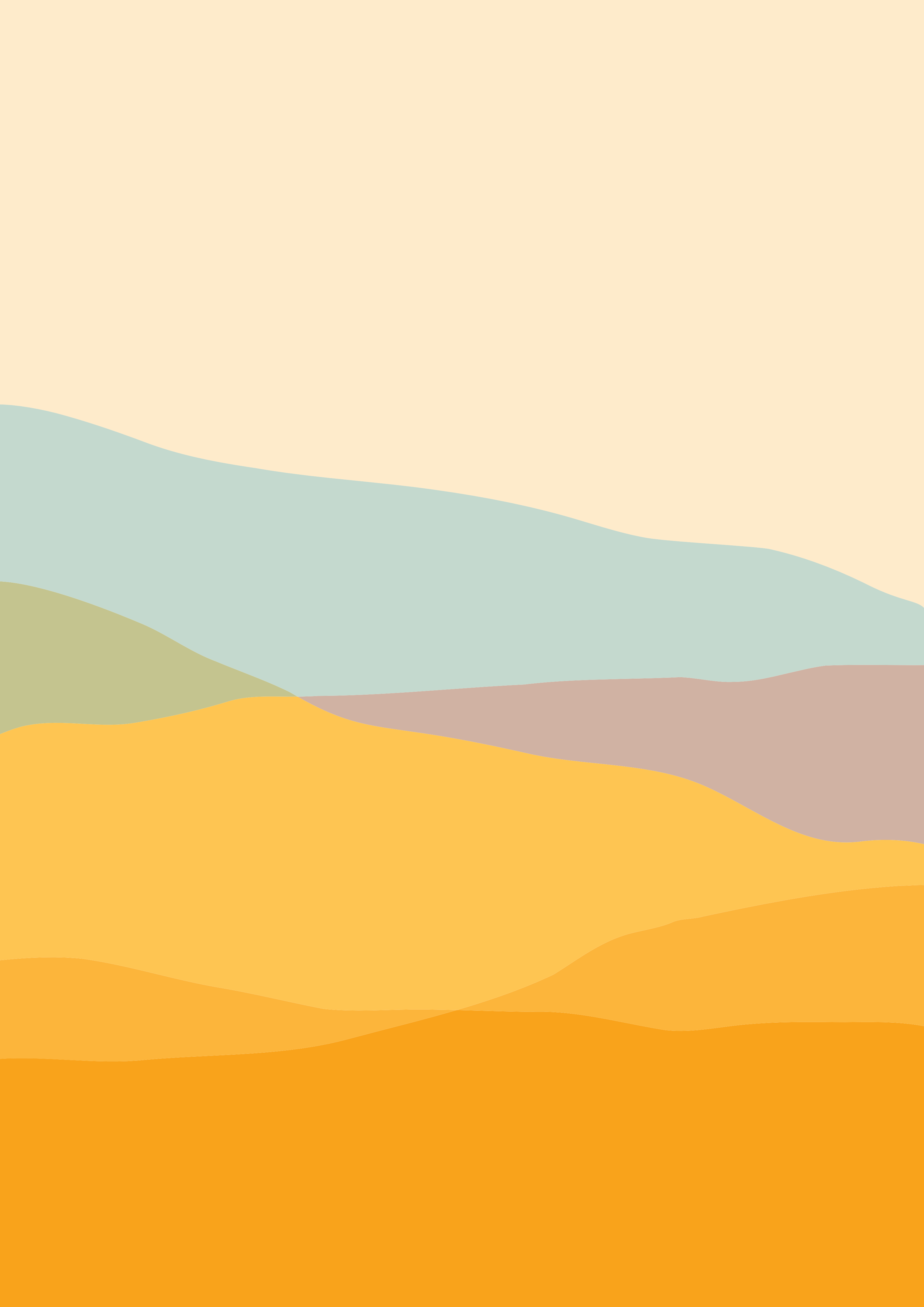

Supplement: Supplementary file 1 — Additional file 1. Study rapid ethnography tools. [file 43058_2023_423_MOESM1_ESM.pdf]
