# Supplementary material for: Rapid ethnography and participatory techniques increase onchocerciasis mass drug administration treatment coverage in Benin: a difference-in-differences analysis
Source: Implement Sci Commun. 2023 Apr 26;4:45. doi: 10.1186/s43058-023-00423-5 (PMC10132427; doi:10.1186/s43058-023-00423-5)
Supplement: Supplementary file 2 — Additional file 2. Coverage survey [file 43058_2023_423_MOESM2_ESM.docx]

**Appendix 2. Coverage survey**

| **Question** | **Response^1^** |
| --- | --- |
| Interviewer name | *[Drop down menu]* |
| Commune | *[Drop down menu]* |
| Arrondissement | *[Drop down menu]* |
| Village | *[Drop down menu]* |
| Household ID | Automatically assigned |
| Head of household name | *[Write-in response]* |
| Has this household consented to participate? | 1, Yes  0, No |
| Please ask the head of household: Has a CDD visited this household in the past several weeks to distribute drugs to your family? | 1, Yes  0, No |
| Please ask the head of household: Did you hear that drug distribution was going to take place before the CDDs arrived at your home? | 1, Yes  0, No  2, A CDD did not come to my home in the past several weeks |
| How did you hear about MDA? | 1, Family member/friend/neighbor  2, Professional health staff  3, Community drug distributor/teacher  4, Community or religious leader  5, Radio  6, Social media  777, Don’t know/Cannot recall  888, Refused to answer  999, Other |
| If other, please explain | *[Write-in response]* |
| Please ask the head of household: When the CDD came to treat your family, did they put a marking of any kind on the outside of your home? | 1, Yes  0, No  2, A CDD did not come to my home in the past several weeks |
| For each family member in the home, please answer the following questions: | |
| Household member’s sex | 0, Male  1, Female  999, Other |
| Household member’s age | *[Write-in response]* |
| Is the household member present? | 1, Yes  0, No |
| Has someone offered the household member a medicine called ivermectin in the last two weeks to treat onchocerciasis, either at home or in the community? (Interviewer should take out the packet of Ivermectin to show the household member) | 1, Yes  0, No  777, Don’t know  888, Refused to answer |
| If no, why was treatment not offered to the individual? | 1, Underage (under 5 years old)  2, Pregnant  3, Breastfeeding  4, Sick  5, Not at home  6, Didn’t hear about MDA  7, Drug ran out  8, Nobody came  999, Other  888, Refused to answer |
| If other, please explain. | *[Write-in response]* |
| Did the household member swallow the ivermectin? | 1, Yes  0, No  777, Don’t know  888, Refused to answer |
| If no, what is the reason the individual did not swallow the drugs? | 1, Fear of side effects  2, Bad taste  3, Not sick  4, Not enough information given by CDDs  5, The drugs don’t work  6, Not eligible for treatment  999, Other  888, Refused to answer |
| If other, please explain. | *[Write-in response]* |
| If yes, what is the reason the individual did swallow the drugs? | 1, Fear of disease(s)  2, To treat disease(s)  3, Because it was given/free  4, Useful information from CDD  999, Other  888, Refused to answer |
| If other, please explain. | *[Write-in response]* |
| If yes, did you swallow the tablet in front of the person who gave it to you? | 1, Yes  0, No  777, Don’t know  888, Refused to answer |
| Do you recall taking this drug (ivermectin) during the drug distribution that took place last year, in 2019? | 1, Yes  0, No  777, Don’t know  888, Refused to answer |
| Please check the outside of the house. Is there a marking left there by the CDD? | 1, Yes  0, No |
| ^1^ Relevance/survey logic not shown |  |
