# Supplementary material for: Rapid ethnography and participatory techniques increase onchocerciasis mass drug administration treatment coverage in Benin: a difference-in-differences analysis
Source: Implement Sci Commun. 2023 Apr 26;4:45. doi: 10.1186/s43058-023-00423-5 (PMC10132427; doi:10.1186/s43058-023-00423-5)
Supplement: Supplementary file 3 — Additional file 3. Dissemination meeting survey [file 43058_2023_423_MOESM3_ESM.docx]

**Appendix 3. Dissemination meeting survey**

| **Measure** | **Question** | **Completely disagree** | **Disagree** | **Neither agree nor disagree** | **Agree** | **Completely agree** |
| --- | --- | --- | --- | --- | --- | --- |
| Acceptability | Using rapid ethnography within NTD programs in Benin is appealing to me. | ① | ② | ③ | ④ | ⑤ |
| Acceptability | I like rapid ethnography as an NTD program strategy in Benin. | ① | ② | ③ | ④ | ⑤ |
| Acceptability | I welcome rapid ethnography within the NTD program in Benin | ① | ② | ③ | ④ | ⑤ |
| Appropriateness | Rapid ethnography to increase coverage of MDA seems like a good match for the Benin NTD program. | ① | ② | ③ | ④ | ⑤ |
| Appropriateness | I believe rapid ethnography could be effective in increase MDA coverage in Benin. | ① | ② | ③ | ④ | ⑤ |
| Feasibility | Rapid ethnography seems possible to implement in Benin. | ① | ② | ③ | ④ | ⑤ |
| Feasibility | Rapid ethnography to increase MDA coverage seems easy to use in Benin. | ① | ② | ③ | ④ | ⑤ |
| Intentions to incorporate | The cost of implementing rapid ethnography seem worth it to me. | ① | ② | ③ | ④ | ⑤ |
| Intentions to incorporate | I believe my team will use the rapid ethnography approach in hard to reach areas. | ① | ② | ③ | ④ | ⑤ |
| Intentions to incorporate | I would like the PACT team to develop and share a rapid ethnography toolkit with my organization | ① | ② | ③ | ④ | ⑤ |
